# Twist1-related miR-26b-5p suppresses epithelial-mesenchymal transition, migration and invasion by targeting SMAD1 in hepatocellular carcinoma

**DOI:** 10.18632/oncotarget.8328

**Published:** 2016-03-24

**Authors:** Yong Wang, Baocun Sun, Xiulan Zhao, Nan Zhao, Ran Sun, Dongwang Zhu, Yanhui Zhang, Yanlei Li, Qiang Gu, Xueyi Dong, Meili Wang, Jindan An

**Affiliations:** ^1^ Department of Pathology, Tianjin Medical University, Tianjin 300070, China; ^2^ Department of Pathology, Tianjin Cancer Hospital, Tianjin Medical University, Tianjin 300060, China; ^3^ Department of Pathology, Tianjin General Hospital, Tianjin Medical University, Tianjin 300052, China; ^4^ Department of Prosthodontics, Affiliated Stomatological Hospital, Tianjin Medical University, Tianjin 300070, China; ^5^ Department of Pathology, Mudanjiang Medical University, Heilongjiang 157011, China

**Keywords:** microRNA, SMAD1, epithelial-mesenchymal transition, early recurrence, hepatocellular carcinoma

## Abstract

Twist1 is well known to induce epithelial-mesenchymal transition (EMT) and promote tumor metastasis. MicroRNAs (miRNAs) are involved in the EMT process and are associated with metastasis in hepatocellular carcinoma (HCC). In the present study, microRNA-26b-5p (miR-26b-5p) expression was consistently and significantly downregulated in HepG2-Twist1 HCC cell lines compared with HepG2-vector cell lines using microarrays (the HepG2-Twist1 cell line can stably express Twist1). miR-26b- 5p downregulation was directly mediated by Twist1 through binding to the promoter region of miR-26b-5p in HepG2-Twist1 cells by ChIP-seq technology. Both gain- and loss-of-function studies showed that miR-26b-5p dramatically suppressed EMT and the invasion ability of HCC cells *in vitro*. Using mouse models, tumors derived from miR- 26b-5p-overexpressed HCC cells exhibited a significant reduction in tumorigenicity compared with the control group. Subsequent investigation revealed that miR-26b-5p directly inhibited SMAD family member 1 (SMAD1) expression. miR-26b-5p repressed BMP4/Smad1 signaling following SMAD1 inhibition. Overexpression of SMAD1 reversed the function of miR-26b-5p. In human HCC tissues and mouse xenograft tumors, miR-26b-5p levels were inversely correlated with SMAD1 expression as well as metastasis. Conclusion: miR-26b-5p suppresses Twist1-induced EMT, invasion, and metastasis of HCC cells by targeting SMAD1 and BMP4/Smad1 signaling. This suggests a promising application for miR-26b-5p in anti-HCC therapy.

## INTRODUCTION

Hepatocellular carcinoma (HCC) is the most common type of primary liver cancer and the third leading cause of death from cancer worldwide. Although promising treatment strategies have been reported, the dismal outcome and poor median survival remain unchanged [1, 2]. The identification of predictive molecular factors and an understanding of the roles of these markers in the molecular genetic mechanisms underlying HCC tumor metastasis would lead to improved overall clinical management of patients with HCC.

MicroRNAs (miRNAs) are endogenously expressed non-coding RNAs that inhibit the expression of their targeted genes. The ability of some miRNAs to function as tumor promoters or suppressors in hepatocarcinogenesis has led to new insights into the molecular pathways involved in HCC [3, 4]. Up to 90% of all human cancers, including HCC, are carcinomas, which are cell growths that originate in epithelial cells. Epithelial-mesenchymal transition (EMT), a normal embryological process, is frequently implicated in cancer aggressiveness and metastasis. It is characterized by loss of cell adhesion, repression of E-cadherin expression, acquisition of the mesenchymal marker vimentin, and increased cell motility and invasiveness [5]. MiRNAs have been demonstrated to exert important regulatory functions in EMT [6].

Twist1, as a b-helix-loop-helix (bHLH) transcription factor, is known to induce EMT in a variety of tumors [7, 8]. In this regulation process, phenotypic plasticity occurs and cell motility is activated [9]. Twist1 can promote migratory and invasive effects on hepatocellular carcinoma by inducing EMT. For HCC patients, high expression and nuclear translocation of Twist1 are important risk factors.

However, the regulatory roles of miRNAs in Twist1-induced EMT contributing to HCC metastasis remain to be elucidated. In this study, we found that Twist1-induced EMT was associated with the downregulation of the miR-26b-5p based on miRNA expression profiles and ChIP-seq technology. Over-expression of miR-26b-5p inactivated the bone morphogenetic protein 4 (BMP4)/Smad1 pathway by upregulating SMAD1 in HCC cells and suppressing EMT, tumor migration and invasion.

## RESULTS

### Expression of miR-26b-5p is inversely correlated with Twist1 expression in HCC tissues and its downregulation is associated with patient poor prognosis

**Figure 1:**
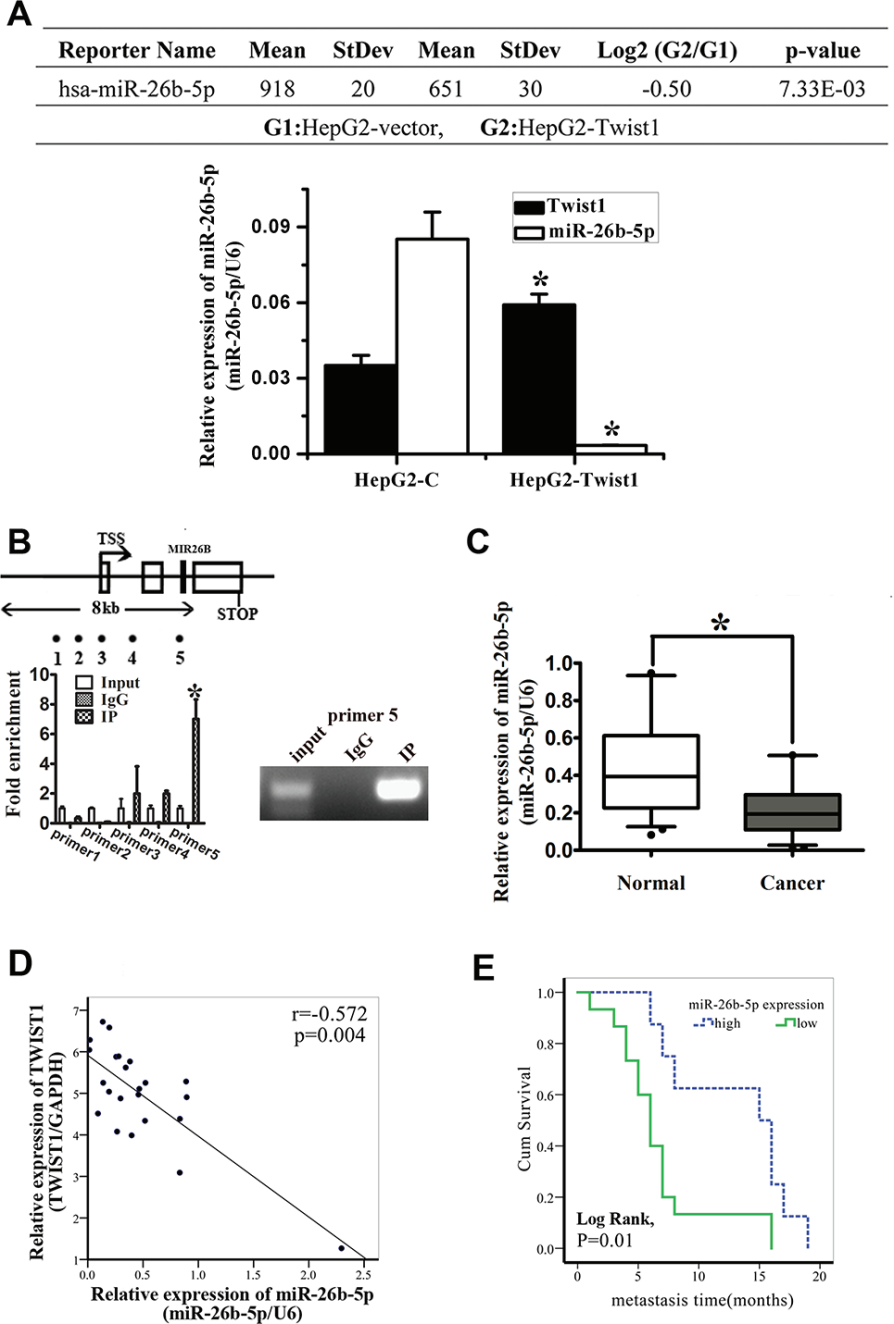
miR-26b-5p is downregulated in HCC tissues and cell lines and is associated with tumor short-term recurrence and metastasis (**A**) Summary of microarray analyses. The upper panel shows that miR-26b-5p is significantly downregulated in HepG2-vector and HepG2-Twist1 human HCC cell lines. The lower panel shows that qRT-PCR was used to verify the results of the chip assay. *P < 0.05. (**B**) ChIP was performed on HepG2 cells transfected with the Twist1 overexpressed plasmid. The precipitated chromatin was PCR-amplified with the use of specific primers in the miR-26b-5p promoter (Table S1) as indicated by black dots. Bar graphs show fold enrichment of Twist1 binding of MIR-26B Transcription Start Site (TSS) region. Relative enrichment compared to irrelative antibody control is shown. The mean ± SD of three determinations is shown. (**C**) Validation of the expression of miR-26b-5p in clinical HCC samples by qRT-PCR. (**D**) An inverse correlation between miR-26b-5p and Twist1 expression using the Pearson correlation (*P* = 0.004, *r* = −0.572). (**E**) Expression of miR-26b-5p is associated with the recurrence and metastasis within the short time of patients with HCC (Log Rank, *P* = 0.01). The average expression value obtained for miR-26b-5p from the 23 samples studied by qRT-PCR was chosen as the cut-off point for survival analysis using the Kaplan-Meier method.

To screen miRNA-related mechanisms underlying EMT followed by the upregulation of Twist1, we performed microarray miRNA analysis on HepG2-vector and HepG2-Twist1 human HCC cell lines. Microarray analysis revealed a panel of miRNAs with significant differential expression between these two HCC cell lines. Among these miRNAs, the expression of miR-26b-5p was significantly (*P* = 0.00733) downregulated in the HepG2-Twist1 cell line compared with the HepG2-vector cell line. The results from microarray studies were subsequently validated by qRT-PCR analysis (Figure 1A). In addition, ChIP-seq analysis indicated that Twist1 could directly bind to the promoter region of miR-26b- 5p and the results were validated by ChIP-qRT-PCR (Figure 1B).

Moreover, the results obtained from HCC patients were consistent with previous observations that the expression of miR-26b-5p was significantly downregulated in HCC tissues (Figure 1C). Pearson correlation analysis showed that miR-26b-5p expression was inversely correlated with Twist1 expression in the clinical samples (Figure 1D). Using the average expression value obtained for miR-26b-5p from the 23 samples studied as the cut-off point for Kaplan-Meier plots, it was demonstrated that lower miR-26b-5p expression was significantly associated with early metastasis of HCC (Figure 1E).

### miR-26b-5p is associated with the EMT phenotype and impairs migratory and invasive abilities in human HCC cell lines

**Figure 2:**
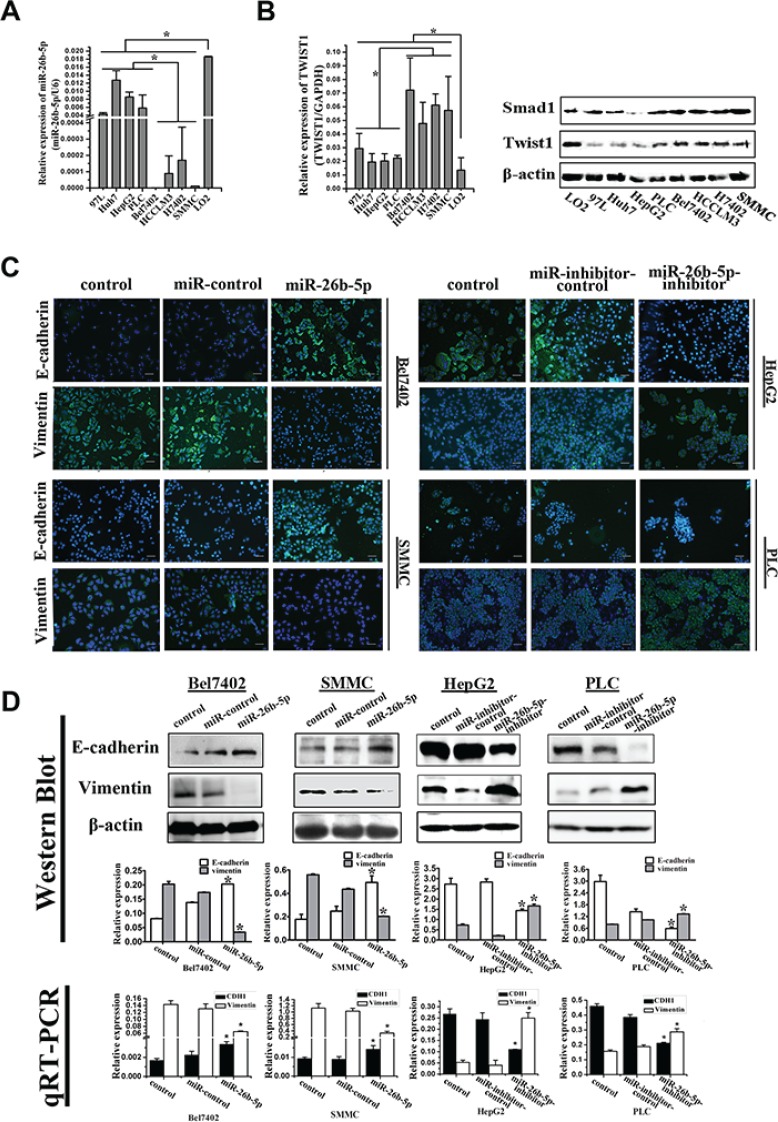
miR-26b-5p is associated with the EMT phenotype in HCC cells (**A**) Expression of miR-26b-5p was studied in a panel of liver cancer cell lines by qRT-PCR. The downregulation of miR-26b-5p was associated with the EMT phenotype: expression of miR-26b-5p was lower in mesenchymal phenotypic cells (Bel7402, H7402, HCCLM3 and SMMC7721) compared to cell lines cells with a basal epithelial phenotype (MHCC97L, HepG2, HuH7 and PLC). (**B**) Expression of Twist1 was studied in liver cancer cell lines by qRT-PCR and Western blot. (**C**) Bel7402 and SMMC7721 cells stably transfected with miR-26b-5p and HepG2 and PLC cells stably transfected with miR-26b-5p-inhibitor were stained for E-cadherin, vimentin, and DAPI and analyzed by confocal microscopy. The green signal represents staining for E-cadherin (upper panel) and vimentin (lower panel). Nuclear DNA was detected by staining with DAPI. Scale bar represents 50 μm. (**D**) Expression of the epithelial protein E-cadherin and the mesenchymal protein vimentin in miR-26b-5p stably transfected Bel7402 and SMMC7721 cells and miR-26b-5p-inhibitor stably transfected HepG2 and PLC cells was detected by Western blot. qRT-PCR was used to detect mRNA expression. β-actin and GAPDH were used as loading controls. Original magnification: 200×. Error bars represent SD and *P < 0.05.

Next, we analyzed the expression of miR- 26b- 5p in a panel of liver cancer cell lines by qRT-PCR. Compared to normal liver cells, the expression of miR- 26b-5p was significantly downregulated in all of the HCC cell lines studied, especially mesenchymal phenotypic HCC cell lines (Figure 2A). The epithelial HCC cells, such as HepG2 and PLC, exhibited high expression of E-cadherin and low expression of vimentin, while HCC cells with a mesenchymal phenotype such as Bel7402 and SMMC7721 demonstrated low expression of E-cadherin and high expression of vimentin (Figure 2C). The data suggest that the expression of the miR-26b-5p may be associated with EMT in HCC.

As miR-26b-5p is a Twist1-related miRNA, the expression of Twist1 was detected in the same HCC cell lines by qRT-PCR and Western blot. Its expression was significantly upregulated in all of the HCC cell lines studied, especially mesenchymal phenotypic HCC cell lines (Figure 2B). This result indirectly shows that lower miR-26b-5p levels were associated with higher Twist1 levels. Thus, we selected and transfected four HCC cell lines as recipient cells: viz.Bel7402 and SMMC7721 with P-miR-26b-5p or P-miR-control, and HepG2 and PLC with P-miR-26b-5p-inhibitor or P-miR-inhibitor-control. Stable cell lines over-expressing and down-regulating miR-26b-5p were established and were tentatively designated Bel7402-miR-26b-5p or SMMC-miR-26b- 5p, HepG2-miR-26b-5p-inhibitor or PLC-miR-26b-5p-inhibitor. The expression of miR-26b-5p in these cells was confirmed by qRT-PCR (Figure S1A and S1B). Compared to P-miR-control transfected cells, upregulation of miR-26b-5p was associated with the observed dramatic morphological changes in the Bel7402-miR-26b-5p and SMMC-miR-26b-5p cells from an elongated fibroblastic phenotype to an epithelial cobblestone phenotype, which is consistent with the changes associated with mesenchymal-to-epithelial transition (MET) (Figure S3A). The reversion of EMT in the Bel7402-miR-26b-5p and SMMC-miR-26b-5p cells was also associated with elevated expression of E-cadherin and the reduced expression of vimentin (Figure 2C and 2D).

**Figure 3:**
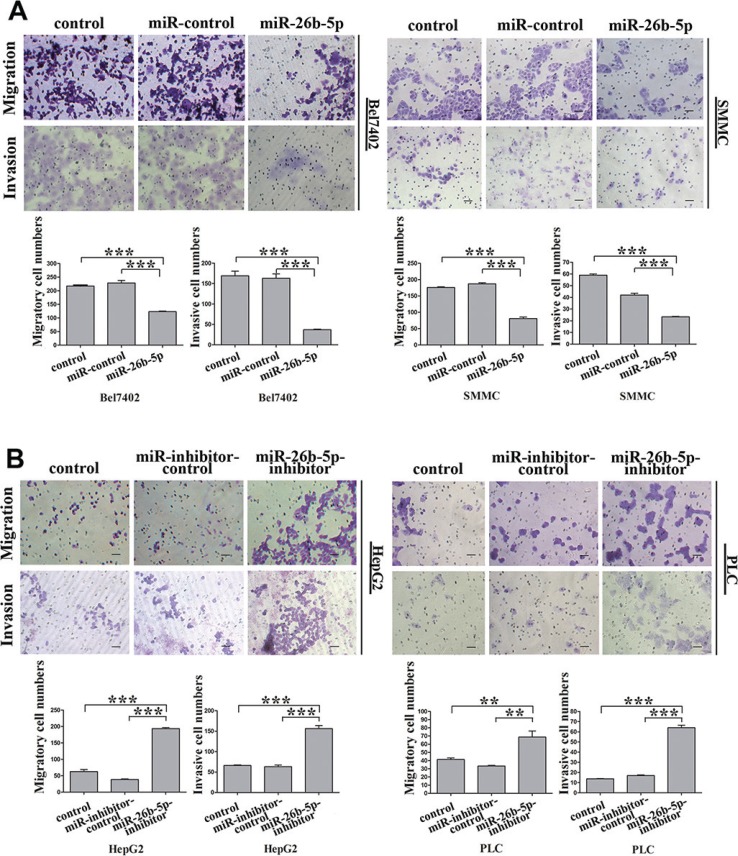
miR-26b-5p alleviates migration and invasion abilities of epithelial HCC cells (**A, B**) Transwell migration and invasion assays with the Bel7402-miR-26b-5p and SMMC-miR-26b-5p cells (A) and HepG2-miR-26b-5p-inhibitor and PLC-miR-26b-5p-inhibitor cells (B). Scale bar represents 50 μm. Migrated cells were plotted as the average number of cells per field of view from three different experiments, as described in the Methods. Original magnification: 200×. Error bars represent SD and *P < 0.05, **P < 0.01, ***P < 0.001.

EMT has been indicated as a key step in initiating cancer cell migration [5]. Therefore, the migration potential of the Bel7402-miR-26b-5p and SMMC-miR-26b-5p cells was examined using transwell migration and invasion assays *in vitro*. The results indicated that stable overexpression of miR-26b-5p significantly alleviated the migration and invasion abilities of the Bel7402 and SMMC cells *in vitro* (Figure 3A). These data indicated that over-expression of miR-26b-5p in HCC with mesenchymal phenotypes inhibited EMT, thus impairing migration and invasion abilities.

HepG2 and PLC cells possess an epithelial phenotype, but following the silencing of miR-26b-5p in HepG2-miR-26b-5p-inhibitor or PLC-miR-26b-5p-inhibitor cells, striking morphological changes consistent with the induction of EMT were observed (Figure S3A). The reduced expression of E-cadherin and the upregulation of vimentin were also observed (Figure 2C and 2D). Furthermore, a significant increase in cell migration and invasion was observed following transfection of p-miR-26b-5p-inhibitor into HepG2 and PLC cells (Figure 3B).

### Stable over-expression of miR-26b-5p suppresses adhesion and colony formation *in vitro* and tumorigenicity *in vivo*

**Figure 4:**
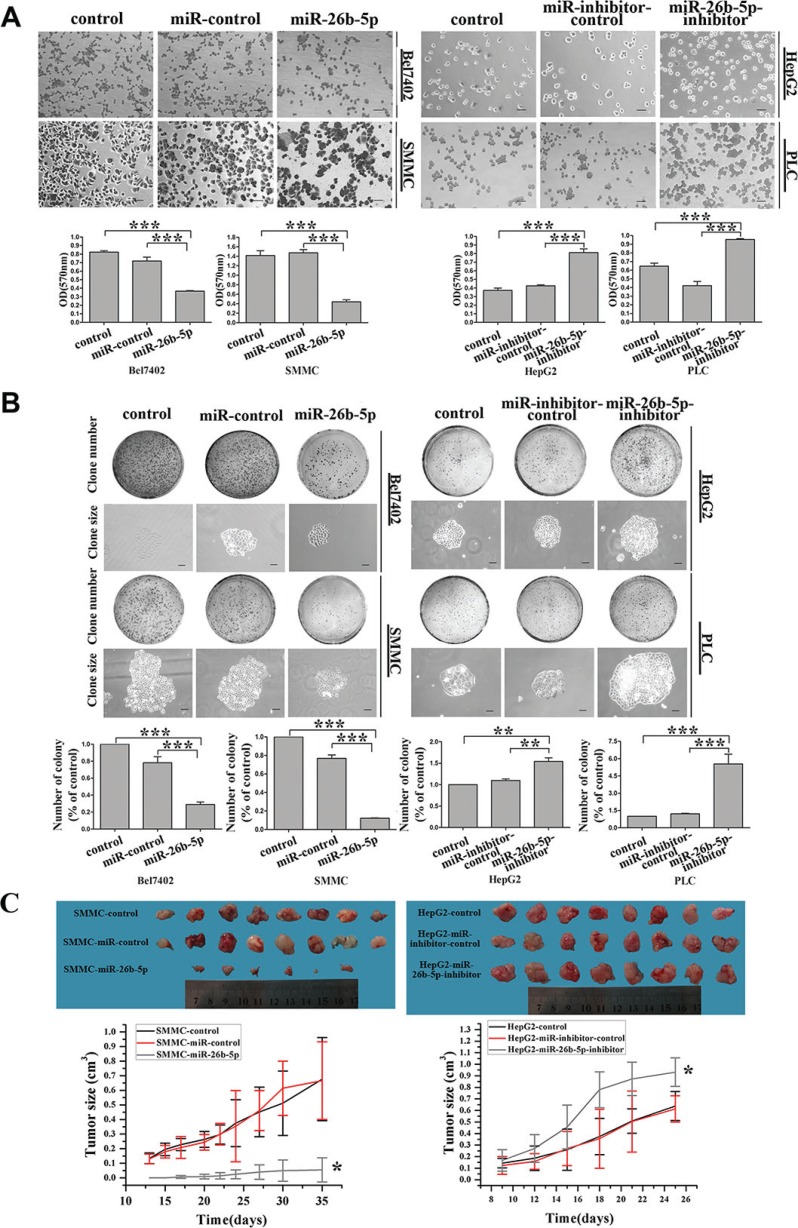
Stable over-expression of miR-26b-5p suppresses adhesive and colony formation abilities *in vitro* and tumorigenicity *in vivo* (**A, B**) Effects of miR-26b-5p on cell adhesion and colony formation of cancer cell lines. Representative results for cell adhesion (A) and colony formation (B) of miR-26b-5p-transfected Bel7402 and SMMC7721 cells and miR-26b-5p-inhibitor-transfected HepG2 and PLC cells. The results were reproducible in three independent experiments. (**C**) SMMC7721 and HepG2 cells (stably transfected with P-miR-26b-5p, P-miR-26b-5p-inhibitor, and their control vectors) were subcutaneously suspended for each mouse. Tumor sizes and growth curves in the treatment and control groups. Error bars represent SD and *P < 0.05, **P < 0.01, ***P < 0.001. Original magnification: 200×, SMMC and HepG2 cells colony formation assay (100×).

EMT program induction follows a well-coordinated process that includes several steps. High cell-matrix interaction is one important step in this program. *In vitro*, cell adhesion assays have emerged as useful tools to evaluate the impact of cell-matrix interaction. Therefore, we evaluated Bel7402-miR-26b-5p and SMMC-miR-26b-5p cells, HepG2-miR-26b-5p-inhibitor, and PLC-miR-26b-5p-inhibitor cells for their adhesion abilities. It was observed that expression of miR-26b-5p suppressed cell adhesion by 49% and 70%, respectively, in Bel7402 and SMMC7721 cells. Inhibition of miR-26b-5p enhanced cell adhesion by 0.86- and 1.14-fold, respectively, in HepG2 and PLC (Figure 4A). This suggests that miR-26b-5p may play an important role in regulating the adhesive traits of HCC cells by reversing EMT.

Next, the capacity for colony formation was evaluated on the cell lines listed above. Interestingly, HepG2-miR-26b-5p-inhibitor and PLC-miR-26b-5p-inhibitor cells generated 2~3-fold more and larger colonies than the corresponding control cells, while Bel7402-miR-26b- 5p and SMMC-miR-26b-5p cells demonstrated opposite results (Figure 4B). These data indicate a growth-inhibitory role for miR-26b-5p *in vitro*.

We further investigated the effects of miR-26b-5p on the tumor growth and metastatic potential of HCC cells using an subcutaneous xenograft tumor model *in vivo*. Five weeks after injection, fewer tumors (75%) were observed in the flanks injected with miR-26b-5p-transfected SMMC7721 cells compared with tumors that appeared at the sites injected with miR-control transfectants in 8 out of 8 (100%) mice. The tumors became palpable 13 to 20 days after inoculation and grew to 22 to 278 mm^3^ by the end of observation (28 days) (Figure 4C). However, miR-26b-5p-inhibitor-transfected HepG2 cells all produced tumors by 10 days after injection, whereas miR-26b-5p-inhibitor-control transfectants produced tumors (mean sizes of 666 to 676 mm^3^ by the end of observation) in all mice (Figure 4C) 9 to 11 days after inoculation. These results indicate that the introduction of miR-26b-5p significantly inhibited tumorigenicity in a nude mouse xenograft model.

Collectively, these findings indicate that miR-26b-5p inhibits both adhesion, colony formation *in vitro* and tumorigenicity *in vivo*.

### SMAD1 is identified as a downstream target of miR-26b-5p

**Figure 5:**
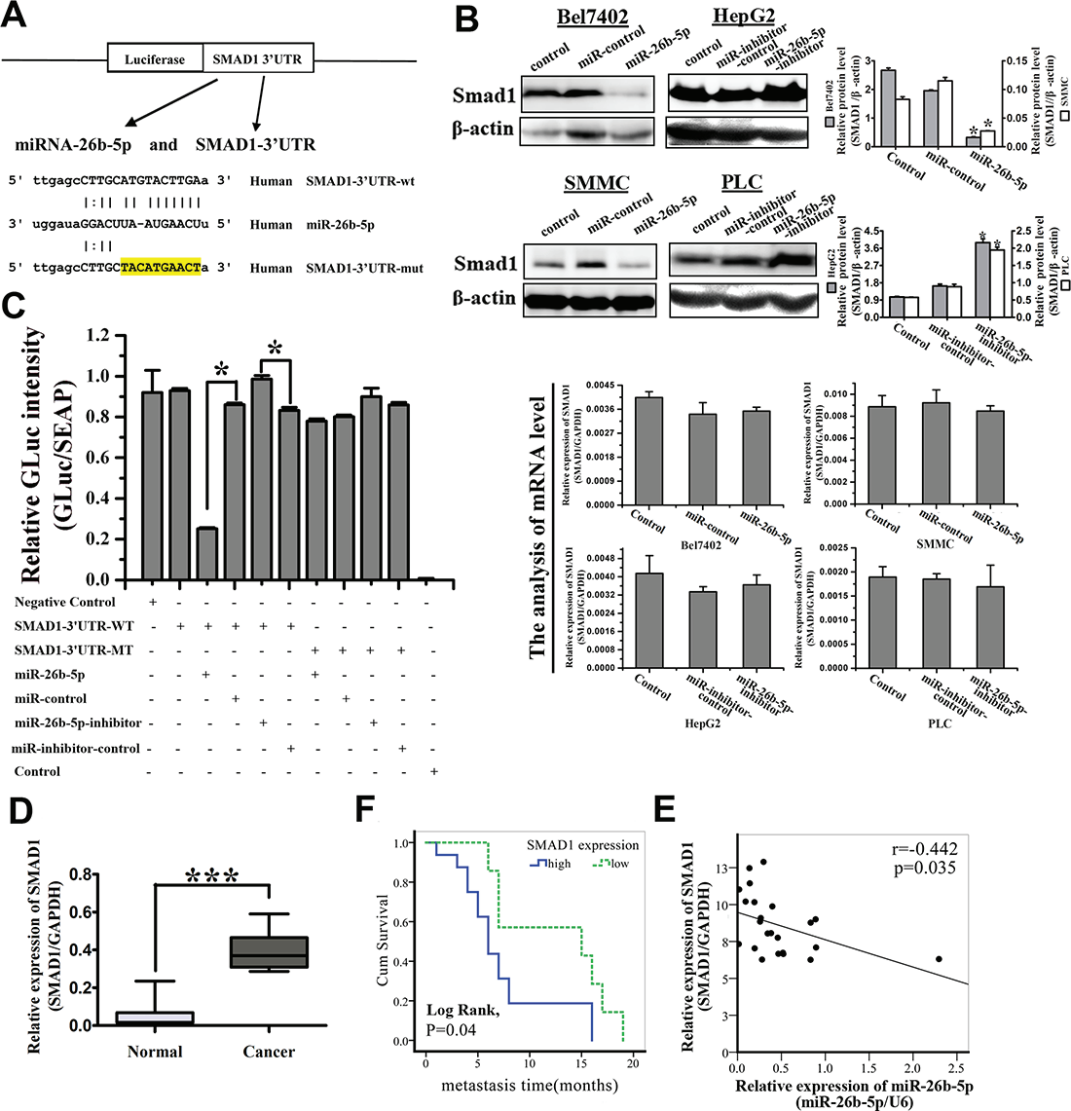
SMAD1 is identified as the functional downstream target of miR-26b-5p and is upregulated in HCC samples (**A**) Sequence alignment of human miR-26b-5p with the 3′-UTR of SMAD1. The seed sequence of miR-26b-5p (middle) matches the 3′-UTR of SMAD1 (top). Bottom, mutation of the 3′-UTR of SMAD1 in the mutant luciferase reporter construct. (**B**) Western blotting (upper panel) and qRT-PCR analysis (lower panel) of SMAD1 expression in P-miR-control- and P-miR-26b-5p-transfected Bel7402 and SMMC7721 cells (left) and P-miR-inhibitor-control and P-miR-26b-5p-inhibitor-transfected HepG2 and PLC cells (right). (**C**) Effects of co-transfection of P-miR-26b-5p with wild-type (wt) and mutant (mut) pGLuc/SEAP-SMAD1 constructs into 293T cells as determined by a dual luciferase assay. Details are described in the Methods. The bar graph shows the mean ± SD in three independent transfection experiments. *P < 0.05. (**D**) The expression of SMAD1 in the same 23 paired HCC tissue samples studied in Figure 1. ***P < 0.001. (**E**) Pearson correlation analysis shows a significant inverse correlation between miR-26b-5p expression levels and SMAD1 mRNA levels in human HCC specimens (*P* = 0.035, *r* = −0.442). The results shown represent the mean ± SD from three independent experiments. *P < 0.05. (**F**) Expression of SMAD1 was associated with the recurrence and metastasis within the short time of patients with HCC (Log Rank, *P* = 0.04). The average expression value obtained for SMAD1 of the 23 samples studied by qRT-PCR was chosen as the cut-off point for survival analysis using the Kaplan-Meier method.

To elucidate the molecular mechanisms by which miR-26b-5p regulates EMT in HCC, we employed several computational algorithms [10] to identify the potential functional targets of miR-26b-5p. Potential target genes of miR-26b-5p were first predicted using databases, including TargetScan, PicTar, and miRanda (Table S4). Among them, SMAD1 was chosen for further experimental validation, not only because it was identified as a target of miR-26b-5p by all three databases, but also due to its well-known importance in both tumor metastasis and EMT. Dual-luciferase reporter analysis showed that expression of miR-26b-5p significantly inhibited the activity of luciferase that carried wildtype but not mutant 3′-UTR of SMAD1 (Figure 5A and 5C), indicating that miR-26b-5p may suppress gene expression through its binding sequence at 3′-UTR of SMAD1. Moreover, introduction of miR-26b-5p in Bel7402 and SMMC diminished the expression of SMAD1 protein, not the expression of SMAD1 mRNA. In comparison, protein expression, not mRNA expression of SMAD1 was significantly increased in HepG2-miR-26b-5p-inhibitor and PLC-miR-26b-5p-inhibitor cells (Figure 5B). These findings indicate that miR-26b-5p may negatively regulate the expression of SMAD1 by directly targeting its 3′-UTR and SMAD1 was indeed a direct downstream target of miR-26b-5p.

Finally, to examine the pathological relevance of this interaction, the expression of SMAD1 was further validated by qRT-PCR in the previous cohort of 23 paired HCC tissues. SMAD1 was demonstrated to be significantly upregulated in HCC samples compared with adjacent histologically normal liver samples (*P* < 0.01) (Figure 5D). The expression of miR-26b-5p and SMAD1 were highly inversely correlated (*P* = 0.035, *r* = −0.442) (Figure 5E). High SMAD1 expression was significantly associated with comparatively early recurrence and metastasis of HCC (Figure 5F). Patients with high expression of SMAD1 had poor prognoses. The data indicate that down-regulation of miR-26b-5p may be responsible for the increased levels of SMAD1 in human HCC tissues, which in turn promotes invasion and metastasis of HCC. Therefore, over-expression of miR-26b-5p inhibits the expression of SMAD1 in HCC cells and high SMAD1 expression correlates with poor prognosis of HCC disease.

### Re-expression of SMAD1 partially abrogated miR-26b-5p-mediated EMT suppressive effects and metastasis suppression

**Figure 6:**
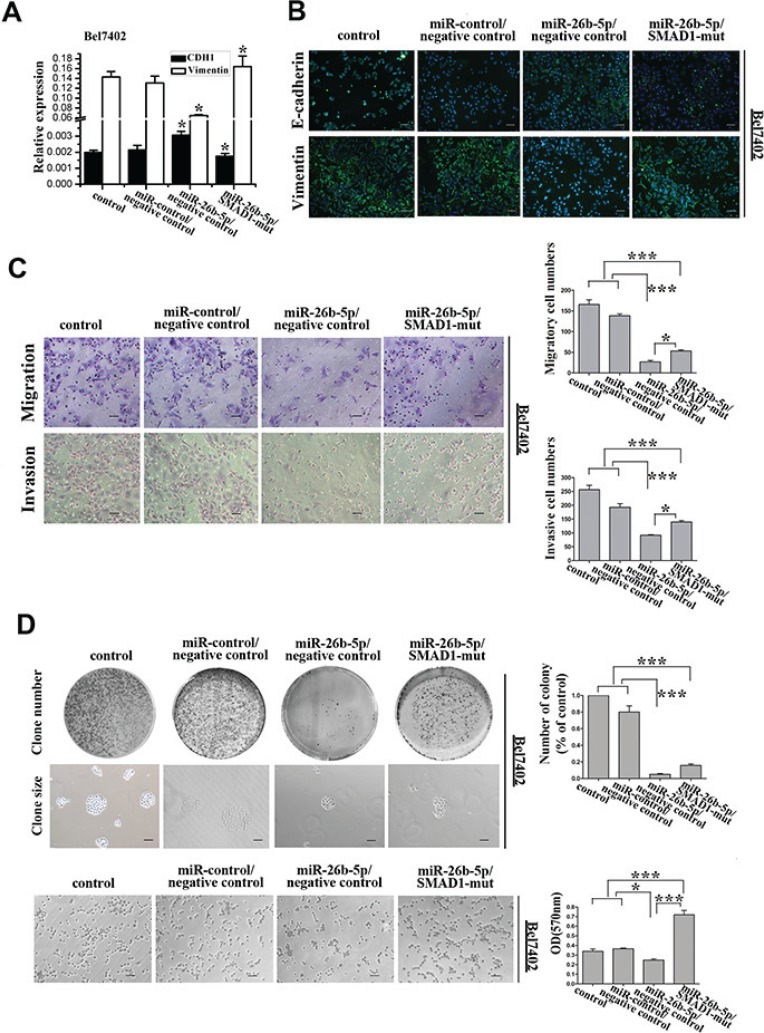
Re-expression of SMAD1 partially rescued miR-26b-5p-mediated EMT, cell migration and invasion abilities in Bel7402-miR-26b-5p cells (**A**) qRT-PCR analysis of the expression of the epithelial protein E-cadherin and the mesenchymal protein vimentin in SMAD1-transfected Bel7402-miR-26b-5p cells. GAPDH was used as a loading control. (**B**) Expression of E-cadherin, vimentin, and DAPI following transfection of SMAD1 into Bel7402-miR-26b-5p cells was analyzed by confocal microscopy. The green signal represents staining for E-cadherin (upper panel) and vimentin (lower panel). Nuclear DNA was detected by staining with DAPI. Scale bar represents 50 μm. (**C**) The migratory properties of the stably transfected cells following the re-expression of SMAD1 in Bel7402-miR-26b-5p cells were analyzed in transwell migration and invasion assays (left). Migrated cells were plotted as the average number of cells per field of view from three different experiments (right), as described in the Methods. (**D**) The colony formation and cell adhesion assays were used to analyze colony formation and cell adhesion abilities. Original magnification: 200×. Scale bar represents 50 μm. The results are summarized in the bar graph. Error bars represent SD and asterisks denote statistical significance < 0.05. ***P < 0.001.

If SMAD1 indeed acts as a functional target of miR-26b-5p, reintroduction of SMAD1 into miR-26b-5p-expressing cells should be able to antagonize the effects of miR-26b-5p. To test this hypothesis, Bel7402-miR-26b-5p cells were transfected with plasmids carrying mutant-type SMAD1 (p-SMAD1-mut), to avoid the influence of the miRNA, into Bel7402-miR-26b-5p cells. Re-expression of SMAD1 in Bel7402-miR-26b-5p cells, as confirmed by qRT-PCR analysis (Figure S2A), induced a dramatic morphological change in the Bel7402-miR-26b-5p cells (Figure S2B), implicating EMT. The induction of EMT observed with p-SMAD1-mut in Bel7402-miR-26b-5p cells was associated with the downregulation of E-cadherin and the increased expression of vimentin (Figure 6A and 6B).

Consistent with these above results, the restoration of SMAD1 inhibited the miR-26b-5p-suppressed migration, invasion, colony formation and cell adhesion (Figure 6C and 6D). These results phenocopied those observed with reduced miR-26b-5p expression.

It is noteworthy that the SMAD1 overexpressing cells exhibited an EMT phenotype and increased aggressiveness compared with the other cells due to the higher expression of SMAD1 in these cells. Taken together, these findings show that SMAD1 reintroduction could partially rescue miR-26b-5p-mediated EMT, cell migration and invasion suppression in HCC cells, suggesting that SMAD1 is a functional mediator of miR-26b-5p in HCC cells.

### MiR-26b-5p inactivates the BMP4/Smad1 pathway and exerts its functions by targeting SMAD1

**Figure 7:**
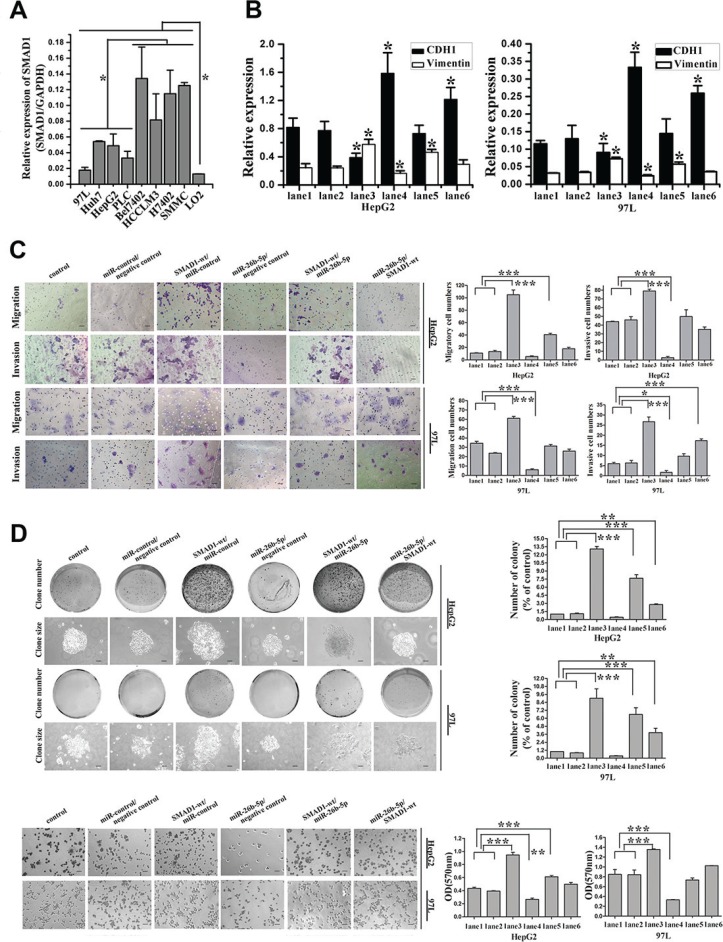
MiR-26b-5p and SMAD1 affect HCC cell migration and invasion by way of regulation of EMT in HepG2 and MHCC97L cells (**A**) Expression of SMAD1 was studied in liver cancer cell lines by qRT-PCR. The protein expression of SMAD1 was studied in liver cancer cell lines by Western blot as in Figure 2B. (**B**) qRT-PCR analysis of the expression of E-cadherin and vimentin in SMAD1 or miR-26b-5p transfected HepG2 and MHCC97L cells. (**C**) The migratory properties of the stably transfected cells following the re-expression of SMAD1 in HepG2 and MHCC97L cells were analyzed by transwell migration and invasion assays. (**D**) Reintroduction of miR-26b-5p antagonized the cell adhesion and colony formation abilities of SMAD1. In (B), (C) and (D), HepG2 and MHCC97L cells were cotransfected with control (lane 1), miR-control/negative control (lane 2), SMAD1-wt/miR-control (lane 3), miR-26b-5p/negative control (lane 4), SMAD1-wt/miR-26b-5p (lane 5), and miR-26b-5p/SMAD1-wt (lane 6). Original magnification: 200×, scale bar represents 50 μm. Colony formation assay (100×), scale bar represents 100 μm. Error bars represent SD and *P < 0.05, **P < 0.01, ***P < 0.001.

To avoid the possible influence of endogenous Twist1 and Smad1, HepG2 and MHCC97L cell lines with medium Twist1 and SMAD1 expression compared with other HCC cell lines (Figures 2B, 7A) were selected. In these two cell lines, we reconfirmed that miR-26b-5p and SMAD1 affected EMT, cell migration, invasion, and adhesion.

First, due to increased miR-26b-5p or the overexpression of SMAD1, or the co-overexpression of miR-26b-5p and SMAD1, a switch in cellular shape between spindle- and fibroblast-like morphologies and the cobblestone-like appearance of epithelial cells was observed (Figure S3B). To determine whether the typical molecular alterations of EMT occurred, we examined the protein and mRNA expression of E-cadherin and vimentin in transfected HepG2 and MHCC97L cells. Interestingly, E-cadherin mRNA expression was up-regulated by 0.9-fold and 1.9-fold whereas vimentin was down-regulated by 31.8% and 23.7% in HepG2 and MHCC97L cells, respectively, following the upregulation of miR-26b- 5p. Additionally, opposing results were observed when SMAD1 was overexpressed (Figure 7B). Moreover, in these two transfected HCC cells, cell migration, invasion, colony formation ability and adhesion ability were enhanced by the overexpression of SMAD1 (Figure 7C, 7D). The increase in miR-26b-5p exerted opposing changes in these assays. The results of qRT-PCR and functional assays fell in between when both miR-26b-5p and SMAD1 were upregulated. These data suggested that miR-26b-5p and SMAD1 influenced the migratory and invasive behaviors of HCC cells by regulating the EMT process.

**Figure 8:**
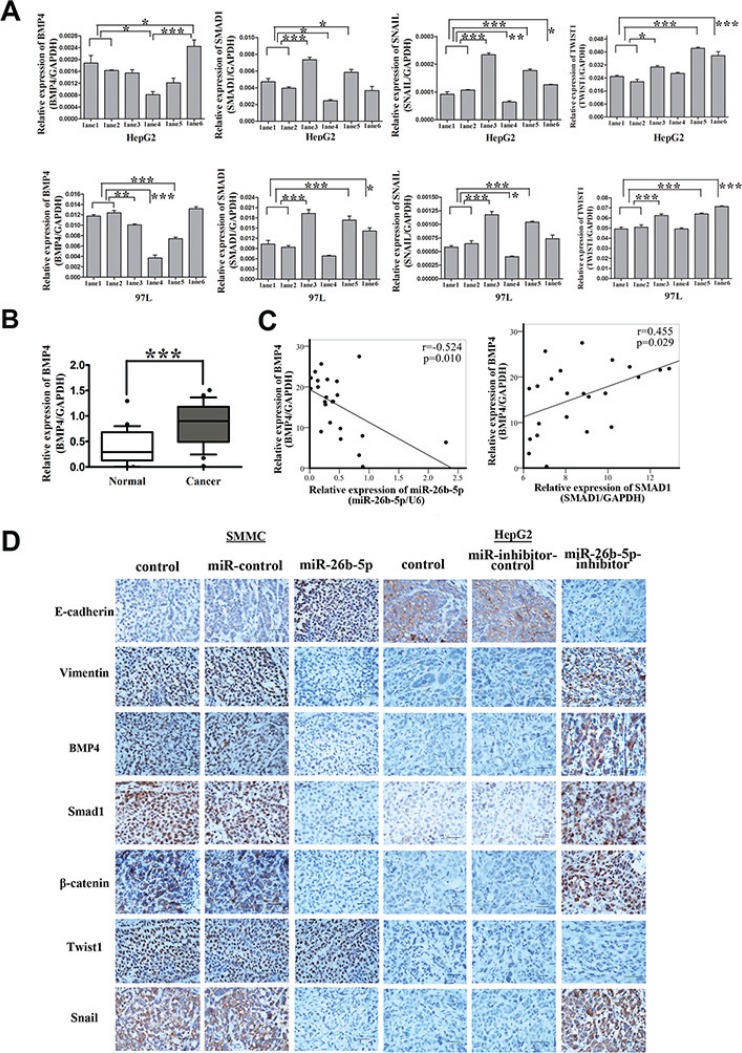
Over-expression of miR-26b-5p inactivates the BMP4/Smad1 pathway to regulate EMT and metastasis by targeting SMAD1 in HCC cells (**A**) qRT-PCR analysis of the expression of BMP4, SMAD1, Snail and Twist1 in SMAD1, miR-26b-5p transfected HepG2 and 97L cells as in Figure 7B. The loading control was GAPDH. (**B**) The expression of BMP4 in the same 23 paired HCC tissue samples studied in Figure 1C. (**C**) Pearson correlation analysis shows a significant inverse correlation between miR-26b-5p expression levels and BMP4 mRNA levels (*P* = 0.01, *r* = −0.524), a significant positive correlation between SMAD1 expression levels and BMP4 mRNA levels (*P* = 0.029, *r* = 0.455) in human HCC specimens. The results shown represent the mean ± SD from three independent experiments. (**D**) Expression levels of EMT-associated markers (E-cadherin, vimentin, BMP4, Smad1, β-catenin, Snail, and Twist1) in engrafted mouse HCC samples in Figure 4C. Original magnification: 400×. Scale bar represents 50 μm. *P < 0.05, **P < 0.01, and ***P < 0.001.

**Figure 9:**
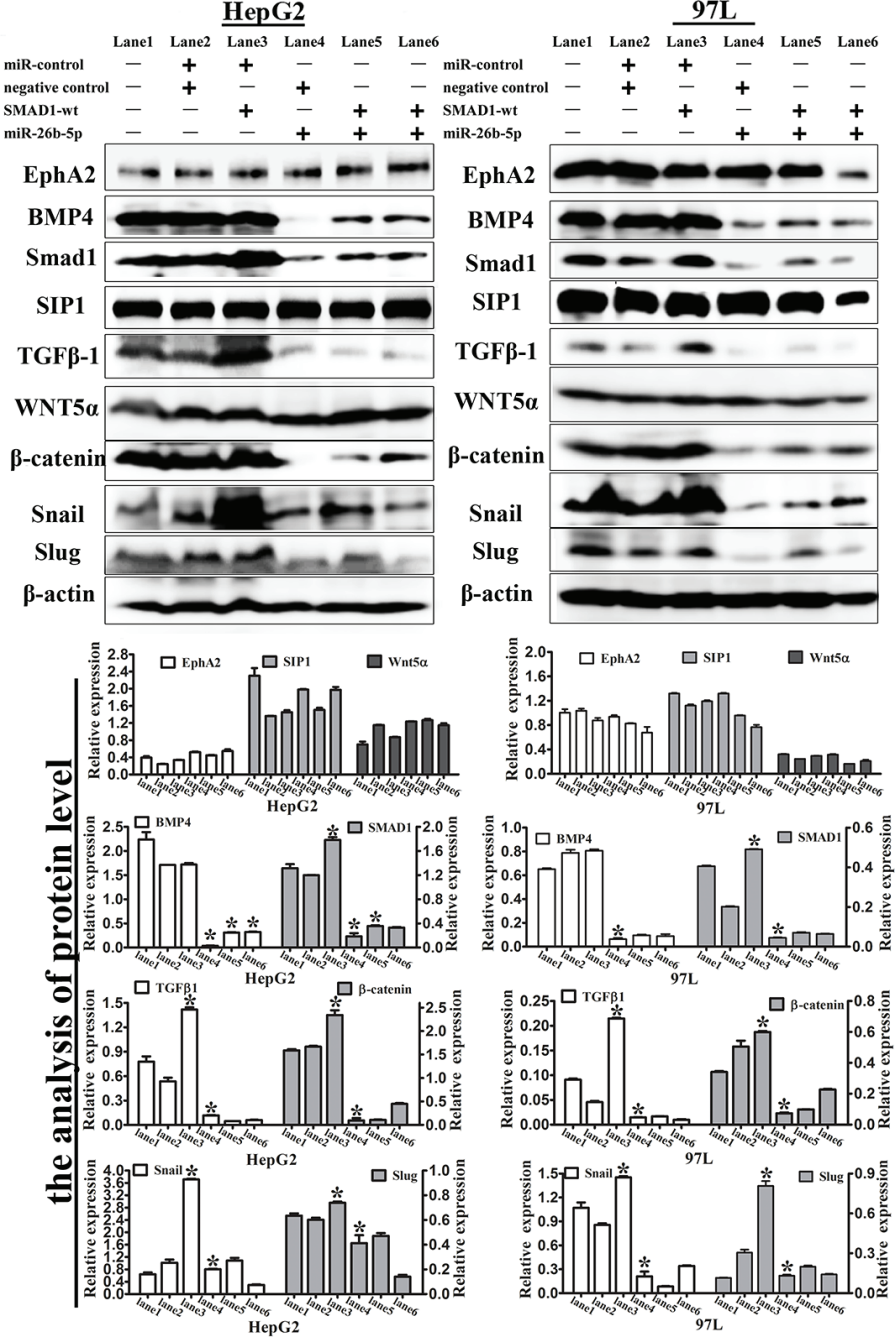
Expression levels of EMT-associated markers in the transfected HepG2 and 97L cells Western blot analysis of the expression of EphA2, BMP4, SMAD1, SIP1, TGFβ1, Wnt5α and β-catenin, Snail, and Slug in SMAD1 or miR-26b-5p transfected HepG2 and 97L cells as in Figure 7B (lane 1: control, lane 2: miR-control/negative control, lane 3: SMAD1-wt/miR-control, lane 4: miR-26b-5p/negative control, lane 5: SMAD1-wt/miR-26b-5p, and lane 6: miR-26b-5p/SMAD1-wt). β-actin was used as a loading control. *P < 0.05.

Smads have been reported to be the central mediators of TGFβ and BMP signaling, which promote tumor invasion and metastasis [11, 12]. Smad1 has been demonstrated in BMP signaling [13–18]. Previous studies have also shown that BMP4 can induce EMT in human cancer cells [19, 20] and that SMAD1 participates in the BMP4-induced EMT process [21, 22]. To evaluate whether the over-expression of miR-26b-5p leading to the downregulation of SMAD1 may inactivate the BMP4 pathway, the expression of BMP4 in the different groups was studied. As expected, downregulation of BMP4 mRNA and protein levels was observed in the miR-26b-5p/negative control (lane 4), SMAD1-wt/miR-26b-5p (lane 5), and miR-26b-5p/SMAD1-wt (lane 6) groups (Figures 8A, 9). The western blot analysis of knockdown of SMAD1 with shRNA in HepG2 and 97L revealed that BMP4 was decreased by downregulation of SMAD1 (Figure S4B and S4C). The results of both overexpression miR-26b-5p and ectopically rescuing expression of SMAD1-mut in Bel7402 showed that the overexpression of SMAD1-mut (Figure S2C) rescued miR-26b-5p-mediated decrease of BMP4 expression. Additionally, the levels of E-cadherin and vimentin in the different groups indicated that miR-26b-5p could inhibit the SMAD1-induced EMT program by downregulating SMAD1 expression (Figure 7B). We further analyzed the expression of EMT-related transcription factors (EphA2, Wnt5α, β-catenin, Snail, Slug, and SIP1/ ZEB2). The data indicated that the levels of EphA2, Wnt5α and SIP1 were not significantly changed in different groups; the expression of β-catenin, Snail, and Slug, was highest in SMAD1-wt/miR-control group, was lowest in miR-26b-5p/negative control group, and in miR-26b-5p/SMAD1-wt group, fell in between (Figures 8A, 9).

The expression of BMP4 was further validated by qRT-PCR in the previous cohort of 23 paired HCC tissues. BMP4 was demonstrated to be significantly upregulated in HCC samples (*P* < 0.01) (Figure 8B). The expression of miR-26b-5p and BMP4 were highly inversely correlated (*P* = 0.01, *r* = −0.524) and there is a significant positive correlation between SMAD1 expression levels and BMP4 mRNA levels (*P* = 0.029, *r* = 0.455) (Figure 8C).

Moreover, we also explored the expression of E-cadherin, vimentin, BMP4, Smad1, β-catenin, Snail, and Twist1 in xenograft mouse models. The results phenocopied the above observations, compared with the controls (Figure 8D, S5).

All together, our data imply that BMP4/Smad1 signaling can be inhibited through SMAD1 repression by miR-26b-5p, thus suppressing EMT, tumor invasion and metastasis.

## DISCUSSION

Here, we demonstrated that there is an important regulatory axis present in certain situations, such as tumor initiation and metastasis, involving high levels of Twist1 binding to miR-26b-5p to downregulate miR-26b-5p expression, resulting in less SMAD1 suppression, inactivation of BMP4/Smad1 signaling, and the promotion of EMT, ultimately contributing to tumor progression in HCC.

The deregulation of miRNAs in HCC and the EMT program has been demonstrated in many reports [6]. Twist1, as an important inducer of EMT and tumor metastasis, is associated with invasion and short survival in HCC patients [7, 23] and has been reported by several groups in HCC patients on GEO database (http://www.ncbi.nlm.nih.gov/geo/). Recent studies have shown that some miRNAs [24–28] can regulate Twist1 or be regulated by Twist1 in human cancers. However, miRNAs that play specific roles in Twist1-induced EMT of HCC have not been well documented. In this study, using microarrays and ChIP-seq technology to analyze Twist1 directly binding to miRNAs, miR-26b-5p drew our attention. Twist1 could directly bind to the promoter region of MIR-26B (Figure 1B). miR-26b-5p is one member of the mature miR-26b family. The miR-26 family is composed of miR-26a-1, miR-26a-2 and miR-26b located on chromosomes 3, 12 and 2, respectively. miR-26 regulates numerous target genes simultaneously and its role is completely different in certain tumors. In glioma, miR-26a has been found overexpressed in a subset of high-grade gliomas and directly targets the PTEN transcript [29]. However, the low expression of miR-26b has been found in glioma cells, and its level is inversely correlated with the grade of glioma [30]. The mature miRNA of miR-26a-1 and miR-26a-2 possesses the same sequence, with the exception of two different nucleotides in mature miR-26b. Thus, the miR-26 family, in part, has similar function, and is consistently down-regulated in a wide range of malignant tumors, including HCC. In HCC, miR-26a has been found to induce a G1 arrest and increase tumor-specific apoptosis [31]. Li et. al. have observed that miR-26b inhibited cell migration and invasion through targeting EphA2 [32]. Additionally, Shen et. al. noted that miR-26b inhibited EMT and acts as tumor suppressor by negatively regulating USP9X in HCC [33]. The low expression of miR-26 is closely related with short overall survival of HCC patients [33–37]. Our clinical data suggested that miR-26b-5p expression was markedly decreased in HCC tissues. Furthermore, downregulation of miR-26b-5p in a panel of liver cancer cells and HCC patients was associated with a more prominent EMT phenotype and poorer prognosis. These clinical observations corroborated well with the *in vitro* and *in vivo* findings reported in the present manuscript using experimental animals and human HCC cell lines. All of the above observations indicate that low levels of miR-26b-5p correlates with Twist1-induced EMT.

By examining the expression of Twist1-related miRNAs between HLME and HLME Twist1-inducible cells after 4 and 12 days of Twist1 induction, an earlier study reported that Twist1-induced miR-424 reversibly drives mesenchymal programming [28]. Instead of the regulation of transient Twist1-induced EMT described above, we used stably Twist1-tranfected HepG2 cells as the treated group in a microarray analysis. Our microarray results showed that miR-424 expression was statistically insignificant (*P* = 0.5856615). Thus, miR-26b-5p, a Twist1 directly-related miRNA, exerts a steady and exclusive role in the EMT program.

EMT is a complex and multi-step program. Cell adhesion, migration, invasion, and proliferation are involved in the EMT process and correlate with tumor metastasis. Therefore, the corresponding functional experiments were performed to explore the possible biological significance of miR-26b-5p in EMT. The results were consistent with our hypothesis that miR-26b-5p impairs these abilities *in vitro*. miR-26b-5p significantly weakened the cell-matrix adhesion both in Bel7402 and SMMC7721 cells. Such activity may diminish the migration and invasion of HCC cells from primary loci and may also result in decreased numbers of HCC cells that colonize target tissues. Based on xenograft mouse models, tumors derived from miR-26b-5p transfectants are obviously smaller than those of the control group. This growth -inhibitory role of miR-26b-5p may result from decreased proliferation. Accordingly, the tumorigenic ability of HCC is suppressed by miR-26b-5p *in vivo*.

SMAD1 has been identified as the putative target for miR-26b-5p using several different miRNA target prediction programs and experimental validation. Our data clearly indicate that miR-26b-5p directly represses invasion and tumor metastasis by inhibiting SMAD1. This conclusion is based on the following evidence. First, miR-26b-5p overexpression significantly decreases the expression of SMAD1 at the protein level in HCC cells. Second, a Dual Luciferase Reporter Assay showed that miR-26b-5p could directly bind to the 3′-UTR of the SMAD1 mRNA. Third, SMAD1 expression is significantly upregulated in HCC tissues, and this up-regulation is strongly correlated with the down-regulation of miR-26b-5p. Fourth, restoration of SMAD1 antagonizes the function of miR-26b-5p. These results indicate that miR-26b-5p directly bind to SMAD1 and down-regulates its expression in HCC.

There are many reports of Smad1 in cancers and its association with advanced cancer stage and metastasis [38–40]. Its expression in HCC patients has been reported by several groups on GEO database (http://www.ncbi.nlm.nih.gov/geo/). In our research, SMAD1 was significantly associated with the early recurrence and metastasis of HCC (Figure 5F) and enhanced cell invasion, migration and cell adhesion, which was the inverse of the functions of miR-26b-5p in HCC cells. A previous study also indicated that SMAD1 participates in the EMT process [22], and the morphologies of the Bel7402 and HepG2 cells changed after the alteration of miR-26b-5p or SMAD1 expression (Figure S3). We speculated that miR-26b-5p and SMAD1 correlate with the EMT process in HCC. As expected, down-regulation of miR-26b-5p or up-regulation of SMAD1 promoted the EMT phenotype. In other words, SMAD1 can increase, whereas miR-26b-5p can suppress, HCC cell migration and invasion by the EMT process. HCC cells with weakened miR-26b-5p or enhanced SMAD1 expression acquire a high adhesive ability. When miR-26b-5p is upregulated, the expression levels of SMAD1 are down-regulated accordingly, and the promotion of the EMT process is relieved. All data indicate that SMAD1 is involved in miR-26b-5p-suppressed EMT process.

SMAD1 belongs to the BMP superfamily of ligands and is involved in complex cell signaling [14–18]. We further explored the pathway by which miR-26b-5p and SMAD1 exert their functions in HCC. The changes in BMP4 expression are very interesting. In the SMAD1-transfected group, BMP4 expression had no statistical significance compared with the control group. Previous experimental data have shown that BMP4 was decreased by downregulation of SMAD1 (Figure S2C, S4B and S4C). However, BMP4 expression in the miR-26b-5p-upregulated group was lower than that in the control group. This indicates that ectopic overexpression of SMAD1 does not increase BMP4 expression and the restoration of SMAD1-mut rescued the miR-26b-5p-suppressed BMP4 expression. A study has shown that PDGF-AA promotes mesenchymal stem cells migration via the BMP-Smad1/5/8-Twist1/Atf4 axis and Twist1 plays the role as a downstream factor of Smad1 [41]. Our data indicated that ectopic overexpression of SMAD1 increases Twist1 expression and when the downregulation of endogenous overexpression of SMAD1 by upregulation of miR-26b-5p expression occurred, Twist1 expression was obviously upregulated (Figure 8A). According to the above study, Twist1 could play its role as a downstream factor of Smad1 and a double-positive feedback loop may exist between SMAD1 and Twist1. As Twist1 directly binds to promoter regions of MIR-26B (Figure 1B) to downregulate miR-26b-5p expression, and SMAD1 has been identified as the target for miR-26b-5p, miR-26b-5p may be an important part in this loop. This hypothesis needs further evidence to be confirmed. Wnt/β-catenin signaling functions upstream of BMP4, and β-catenin can activate the promoters of BMP4 [38]. Thus, in our study, the changes in β-catenin expression were not induced by the activation of Wnt/β-catenin signaling, and β-catenin may be only an EMT-related transcription factor. As we previously stated, SMAD1 has been shown to be present at the crossroads of multiple signaling pathways, such as TGFβ signaling, BMP signaling, Wnt/β-catenin signaling, and others [42–44]. Changes in TGFβ1 expression confirmed the existence of this crossroads in our study. Various publications have shown that BMP-activated Smad signaling is very important for migratory processes. In HCC, BMP4 expression levels are increased and important for carcinogenesis [45, 46]. In our research, BMP4 expression was upregulated in HCC samples. This is consistent with earlier research results. Snail is well known as an EMT inducer and promotes metastatic and tumorigenic abilities. Smad1 as an upstream factor regulates Snail-induced PI-3 kinase/Akt and Nanog expression [47]. Taken together, miR-26b-5p primarily inhibits BMP4/Smad1 signaling by down-regulating SMAD1 expression, thus demonstrating its indispensable role. The results from xenograft mouse models partially phenocopied the above observations, compared with controls.

Additionally, another study found that Slug is a direct transcription target of Twist1 in the repression of E-cadherin during EMT, and these two transcription factors cooperatively promote EMT in human breast tumors [48]. In our study, due to miR-26b-5p up or downregulation, Twist1 expression demonstrated no significant change between the treatment group and control group (Figure S4). Nevertheless, down-regulation of miR-26b-5p or up-regulation of SMAD1 could enhance Slug expression. Moreover, in the microarray analysis, Slug was excluded from miR-26b-5p potential targets. All of the observations indicate that Slug may be only a downstream factor of Smad1 in this study.

In summary, we found that miR-26b-5p deregulation correlates with Twist1-induced EMT. The overexpression of miR-26b-5p inhibited EMT, migratory and invasive abilities, and adhesion by inactivating the BMP4/Smad1 signaling pathway through the downregulation of SMAD1. These results may provide novel prognostic and predictive factors for HCC disease and the design of novel miRNA-based therapeutic strategies against HCC.

## MATERIALS AND METHODS

### Cell culture and transfection

The following cell lines were obtained from the American Type Culture Collection and from Zhongshan Hospital Affiliated to Fudan University (Shanghai, China) in 2014: H7402, Bel7402, SMMC7721, HepG2, PLC, HCCLM3, MHCC97L, Huh7, LO2, and 293T. The various HCC cell lines were cultured in Dulbecco's modified Eagle's medium (DMEM) (Hyclone) with 10% fetal bovine serum (Hyclone). Vectors were transfected into cells via percutaneous ethanol injection (Polysciences, Inc., Cat #23966).

### RNA extraction and microarray analysis

Total RNA was extracted using Trizol reagent (Tiangen Biotech, Beijing, China), and miRNAs were obtained using the miRcute miRNA isolation kit (DP501) (Tiangen Biotech, Beijing, China). miRNA microarray was performed by a service provider (LC Sciences, Houston, TX, USA). The microarray data has been deposited on NCBI GEO database and the GEO number is GSE77501. miRNAs with *p*-values below a critical *p*-value (typically 0.01) were selected for further analysis. Data filtering removed miRNAs with (normalized) intensity values below a threshold value of 32 across all samples. Details of the analysis process were provided in the Supplementaryary materials and methods.

### Chromatin immunoprecipitation (ChIP) assay

Detailed procedures for ChIP assay were described in detail in the Supplementaryary materials and methods. Antibodies against Twist1 (SC-15393 X, Santa Cruz, USA), IgG (AB171870, Abcam, UK) were used in this experiment (5 μg). Antibody-protein-DNA complexes were isolated by immunoprecipitation with protein A agarose beads (GE Healthcare, UK). Following extensive washing, bound DNA fragments were eluted and analyzed by subsequent qRT-PCR. Primer sequences are listed in the Table S1.

### Clinical HCC specimens

Through the Tumor Tissue Bank of Tianjin Cancer Hospital, 23 paired tissue specimens were obtained from 23 patients who underwent hepatectomy for HCC in 2014. The diagnoses of these HCC samples were verified by pathologists. Detailed pathologic and clinical data were collected for all samples including Edmondson tumor grade and metastasis. The use of these tissue samples in this study was approved by the institutional research committee.

### Vector construction

Plasmid pcDNA3-Twist1 has been described. [23] The human pre-miR-26b-5p gene expression plasmid, miR-26b-5p gene expression inhibitor plasmid and the respective empty vector plasmids were purchased from GeneCopoeia (US). Puromycin is the stable cell line selection marker.

The target SMAD1-3′-untranslated region(UTR)-wild and SMAD1-3′UTR-mutant gene expression plasmids (p-SMAD1-3′UTR-wt and p-SMAD1-3′UTR-mut) were purchased from GeneCopoeia (US). The stable cell line selection marker is neomycin.

The small interfering RNA (siRNA) kit (pGP-SMAD1-shRNA) (defined as sh-SMAD1) was purchased from GeneCopoeia (US). Puromycin is the stable cell line selection marker.

### Quantitative real-time polymerase chain reaction (qRT-PCR)

qRT-PCR was performed as previously described [49], using the primers listed in Tables S1, S2. PCR was conducted in a 7500HT Real-Time PCR System (Applied Biosystems, Foster City, CA). A U6 or GAPDH internal control was used as an endogenous control, and fold changes were calculated via relative quantification (2^−ΔCt^ or 2^−ΔΔCt^) [50].

For miRNA detection, a two-step quantitative RT-PCR reaction using the miRcute miRNA cDNA Synthesis Kit (KR201) and miRcute miRNA qPCR Detection Kit (SYBR Green) (FP401) (Tiangen Biotech, Beijing, China) according to the manufacturer's instructions was used. For mRNA detection, qRT-PCR was performed exactly as previously described [49].

### miRNA target prediction

The analysis of miR-26b-5p predicted targets was performed using TargetScan, PicTar, the miRanda algorithms and the complementary DNA (cDNA) microarray results for hepatocellular carcinoma in our previous study. The related functions of the targets were also considered in Kyoto Encyclopedia of Genes and Genomes (KEGG).

### Luciferase reporter assay

The 293T cells were transiently cotransfected with P-miR-26b-5p, P-miR-control, P-miR-26b-5p-inhibitor, P-miR-inhibitor-control, pGluc/SEAP-SMAD1–3′UTR-wt, pGluc/SEAP-SMAD1–3′UTR-mt, and pGluc/SEAP-vector. After 60 hours, the cell culture medium was collected and luciferase activities were analyzed by Secrete-Pair^™^ Dual Luminescence Assay Kits (GeneCopoeia^™^). The results were obtained from three independent experiments performed in duplicate.

### Western blot analysis

Protein extractions and western blot analyses were performed as described. [23] Blots were blocked and incubated with antibody (Table S3).

### Cell adhesion, colony formation and migration/invasion assays

For adhesion assay, transfected Bel7402, SMMC7721 (2.5 × 10^5^/well) or HepG2, and PLC (5 × 10^5^/well) cells were added into 96-well plates coated with Matrigel (0.2 mg/mL). The above transfected cells were seeded in a 6-well plate at a cell density of 1, 000–5, 000 cells/well in colony formation assays. Experiments were performed as previously described [51]. Migration/invasion assays were performed as reported [23].

### Immunohistochemical and immunofluorescence staining

For immunohistochemical staining (IHC), sections were pretreated by microwaving, blocked, and incubated using a series of antibodies (Table S3). For the immunofluorescence assay (IF), the coverslips with cells were incubated with primary antibodies (E-cadherin 1:50, vimentin 1:200). The IHC staining systems and IF experiments used in this study were performed as previously described [23].

### Animal studies

*In vivo*, 5 × 10^6^ SMMC7721 and 1 × 10^7^ HepG2 cells (stably transfected with P-miR-26b-5p, P-miR-26b-5p-inhibitor, and their control vectors) were suspended for each mouse. Each nude mouse (8 in each group, female BALB/c-nu/nu at 3–4 weeks of age) was subcutaneously injected in the upper right flank region at 0.2 mL/mouse with a microsyringe under anesthesia. After 4 or 5 weeks, mice were sacrificed and tumors were harvested and fixed with phosphate-buffered neutral formalin and prepared for standard histological examination. All studies were performed under the American Association for the Accreditation of Laboratory Animal Care guidelines for the humane treatment of animals and adhered to national and international standards.

### Statistical analysis

Data are presented as the mean ± standard deviation (SD). All statistical analyses were performed using ANOVA or a two-tailed Student's *t*-test to compare data. The survival curves were calculated using the Kaplan-Meier method. *P* < 0.05 was considered statistically significant.

## SUPPLEMENTARY MATERIALS FIGURES AND TABLES



## Abbreviations

HCC: hepatocellular carcinoma
EMT: epithelial-mesenchymal transition
E-cadherin: epithelial-cadherin
GAPDH: glyceraldehyde 3-phosphate dehydrogenase
SMAD1: SMAD family member 1
BMP4: bone morphogenetic protein 4
