# Supplementary material for: Twist1-related miR-26b-5p suppresses epithelial-mesenchymal transition, migration and invasion by targeting SMAD1 in hepatocellular carcinoma
Source: Oncotarget. 2016 Mar 24;7(17):24383–401. doi: 10.18632/oncotarget.8328 (PMC5029709; doi:10.18632/oncotarget.8328)
Supplement: Supplementary file 1 [file oncotarget-07-24383-s001.pdf]

Twist1-related miR-26b-5p suppresses epithelial-mesenchymal transition, migration and invasion by targeting SMAD1 in hepatocellular carcinoma

Supplementary Materials

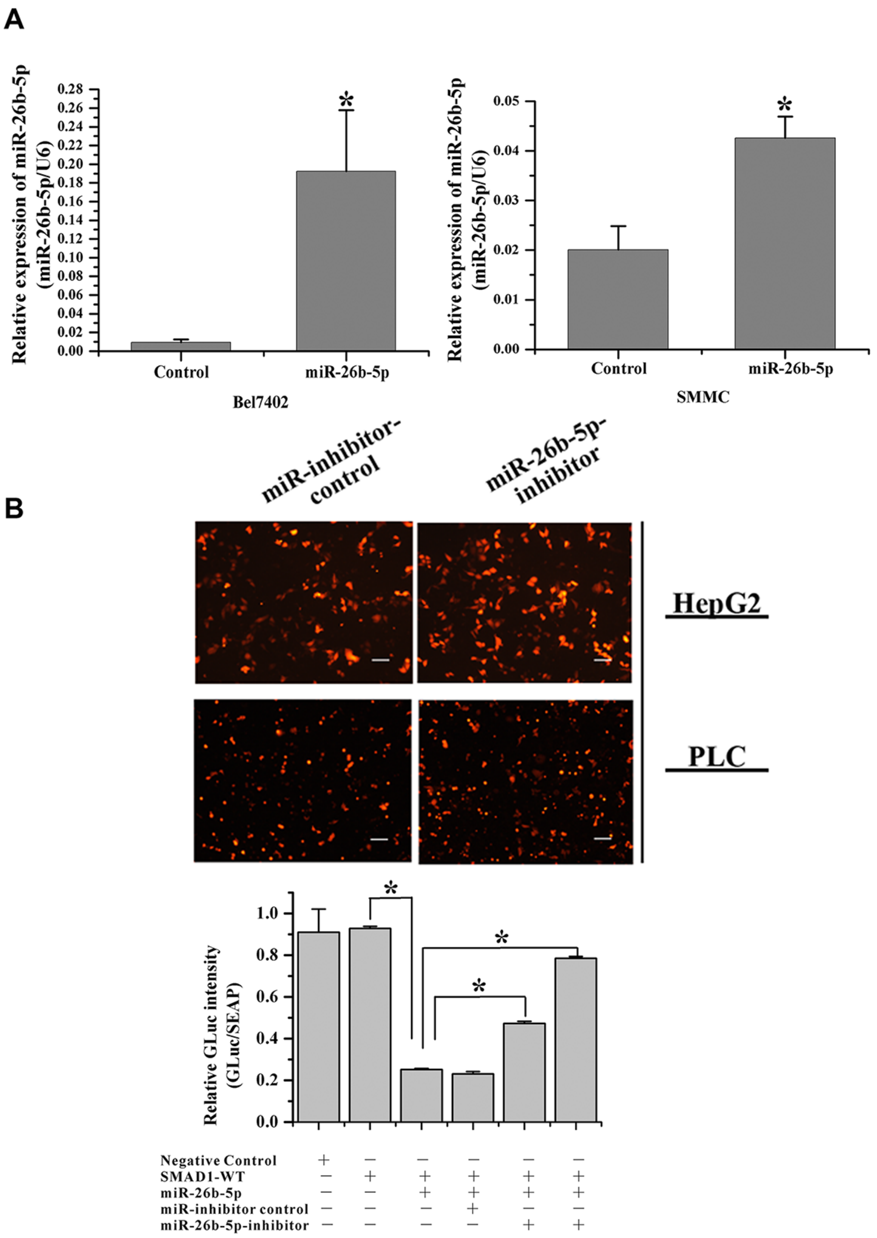

Supplementary Figure S1: The efficiencies of the P-miR-26b-5p and P-miR-26b-5p-inhibitor plasmids. (**A**) qRT-PCR was employed to validate the significant increase of miR-26b-5p in stably-transfected Bel7402-miR-26b-5p and PLC-miR-26b-5p cells, designated as Bel7402-miR-26b-5p or SMMC-miR-26b-5p. (**B**) HepG2 and PLC were transfected with P-miR-26b-5p-inhibitor or P-miR-inhibitor-control, designated as HepG2-miR-26b-5p-inhibitor or PLC-miR-26b-5p-inhibitor. The upper panel shows the transfection efficiency of the P-miR-26b-5p-inhibitor plasmid. Scale bar represents 100 µm. Original magnification: 100×. The lower panel shows that a dual luciferase assay was used to verify the function of the plasmid. The construction of the two plasmids was successful. **P* < 0.05.

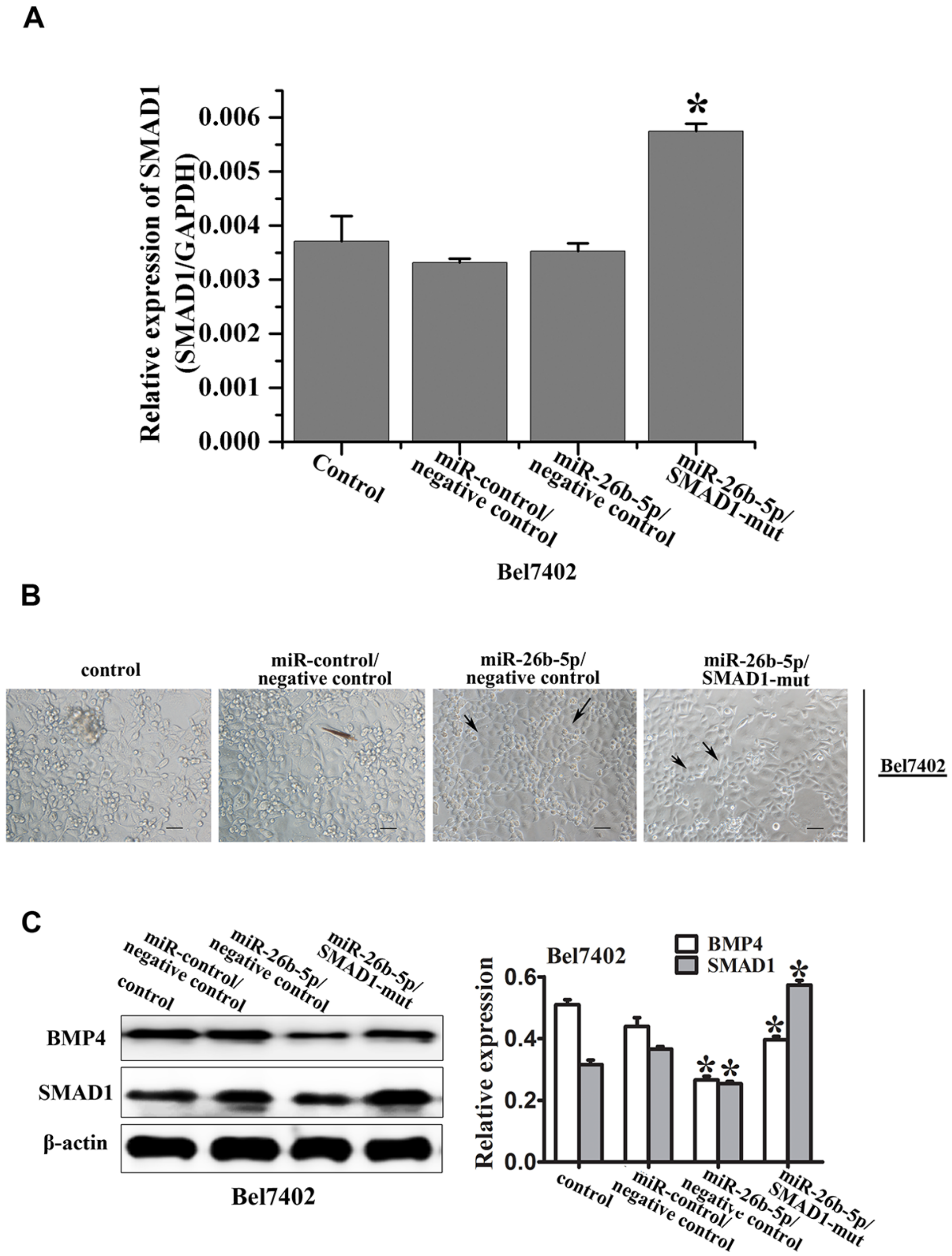

Supplementary Figure S2: (**A**) qRT-PCR was used to confirm the re-expression of SMAD1 in Bel7402-miR-26b-5p cells. *$P < 0.05$. (**B**) Ectopic expression of SMAD1 in Bel7402-miR-26b-5p cells induced a dramatic morphological change from an epithelial cobblestone phenotype to an elongated fibroblastic phenotype, which is consistent with EMT. Scale bar represents 50 µm. Original magnification: 200×. (**C**) Western blotting analysis of BMP4 expression and SMAD1 expression in P-miR-26b-5p- and P-miR-26b-5p/P-SMAD1-mut– co transfected Bel7402 cells. The representative image with quantitative results was provided in the right panel. *$P < 0.05$.

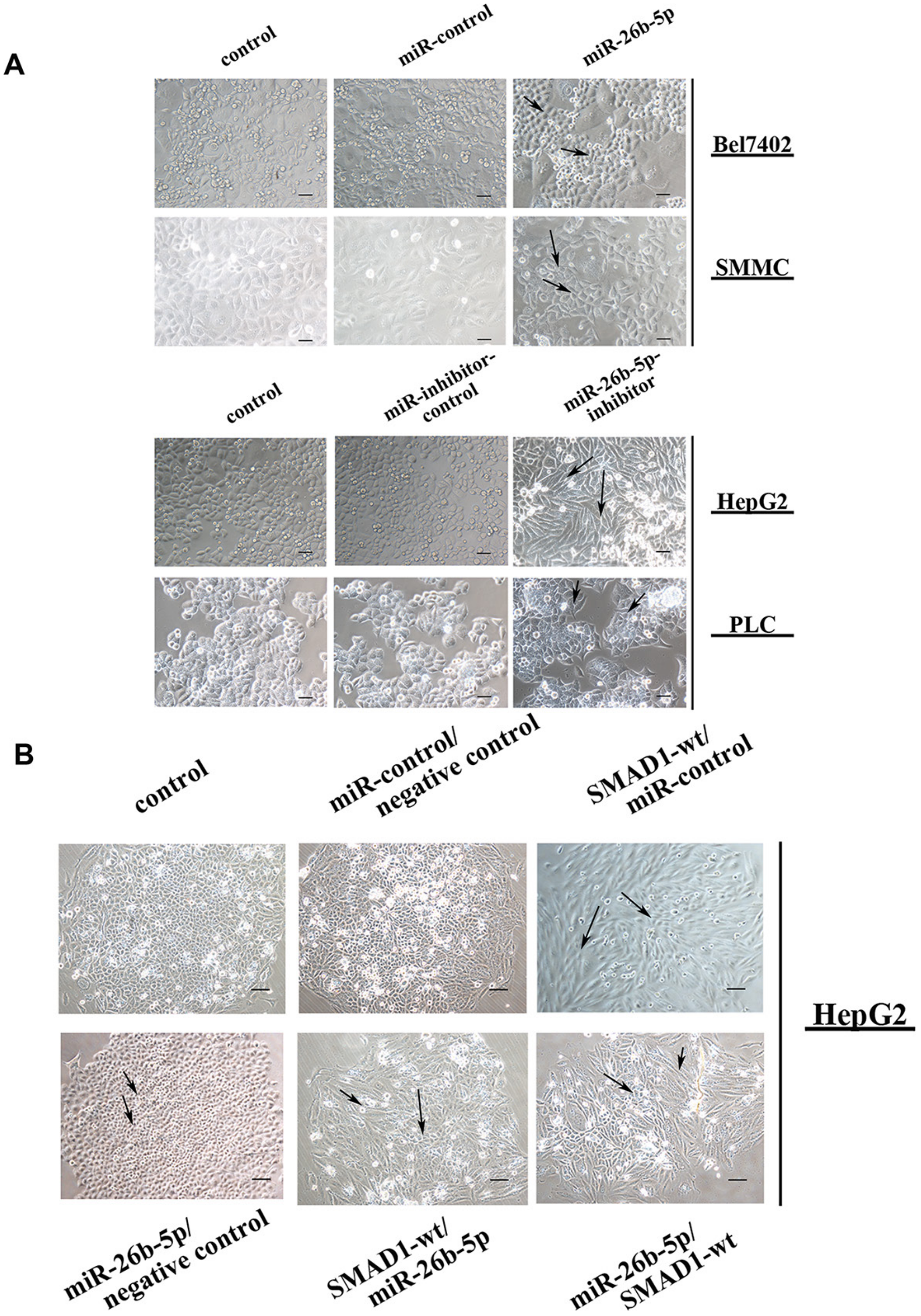

Supplementary Figure S3: (**A**) HepG2-and PLC-miR-26b-5p-inhibitor stably transfected cells demonstrated significant morphological changes from an epithelial cobblestone phenotype to an elongated fibroblastic phenotype, which is indicative of EMT. Bel7402 and SMMC with upregulated miR-26b-5p show the reverse change, as in MET. Scale bar represents 50 µm. Original magnification: 200×. (**B**) With increased miR-26b-5p or the overexpression of SMAD1, or co-overexpression of miR-26b-5p and SMAD1, a switch between the EMT phenotype and MET phenotype was observed. Scale bar represents 100 µm. Original magnification: 100×.

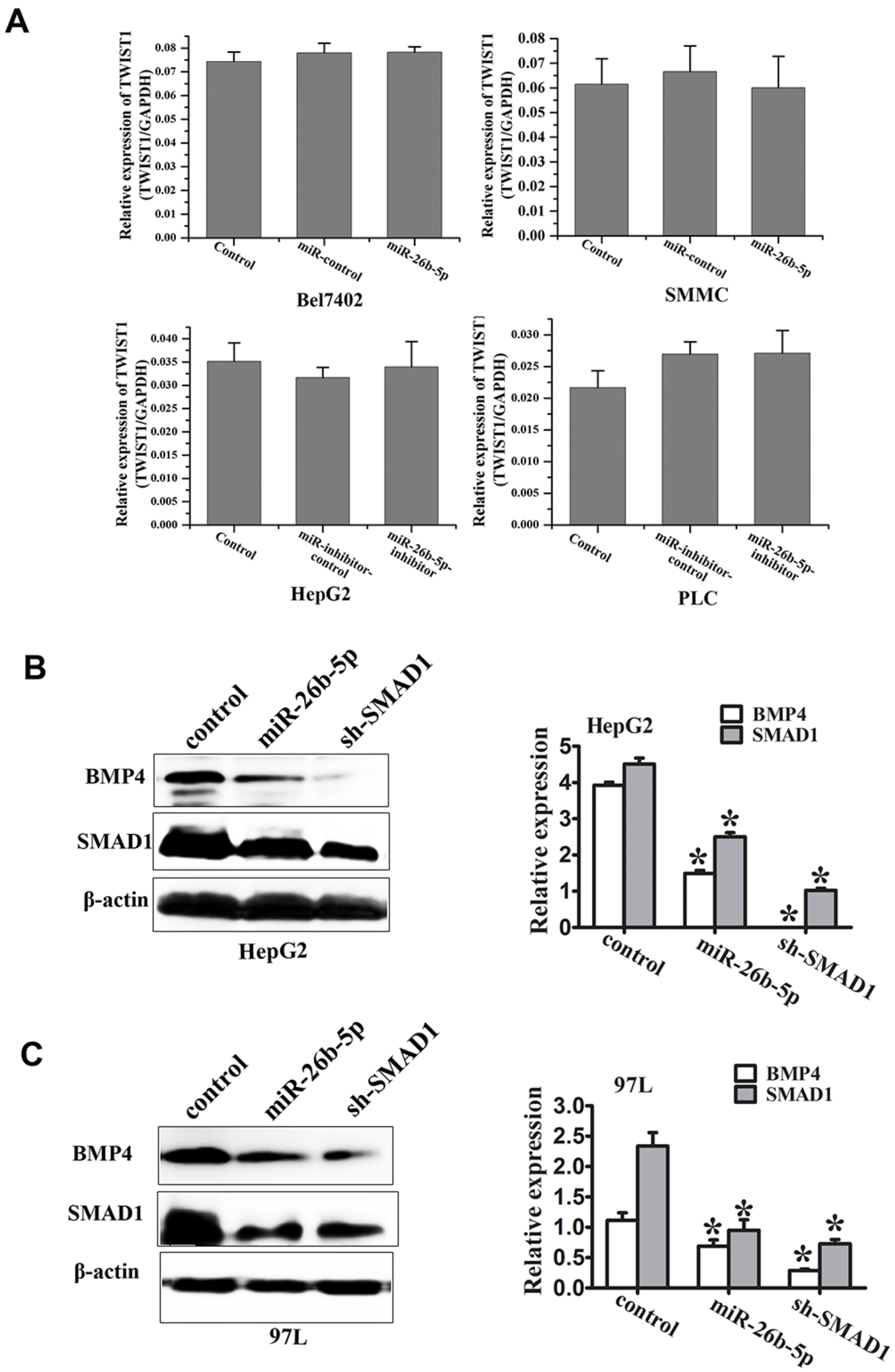

Supplementary Figure S4: (**A**) The Twist1 expression levels in tranfected-Bel7402, SMMC, HepG2 and PLC cells were detected by qRT-PCR. Data represent the means of three separate experiments ± SD. (**B**) (**C**) Western blotting analysis of BMP4 expression and SMAD1 expression in P-miR-26b-5p- and P-SMAD1-shRNA- transfected HepG2 (B) and 97L cells (C). $^*P < 0.05$.

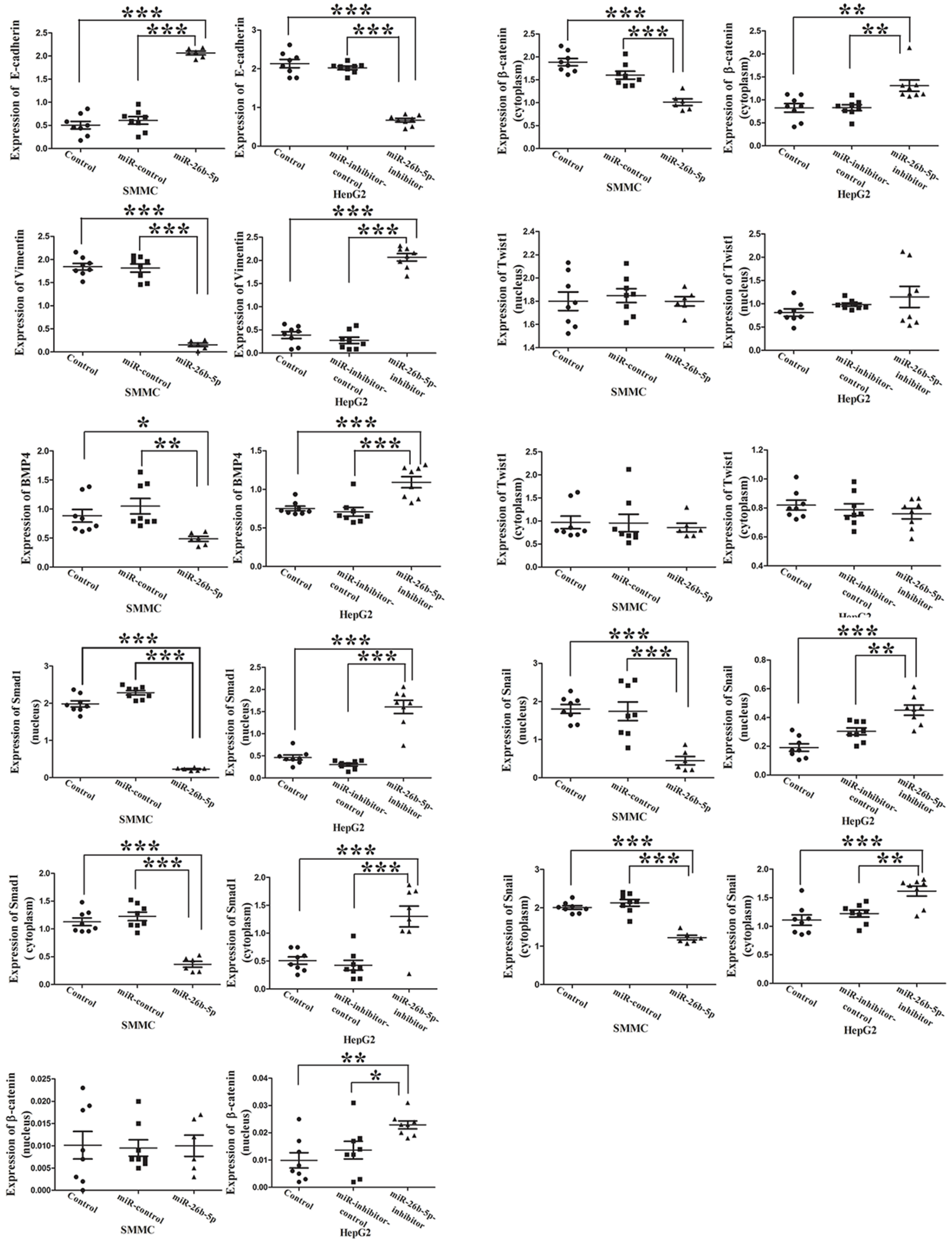

Supplementary Figure S5: Relative expression levels of EMT-associated markers in engrafted mouse HCC samples. Scatter plots were performed to show the expression levels of E-cadherin, vimentin, BMP4, SMAD1, β-catenin, Snail, and Twist1 in engrafted mouse HCC samples by Data represent the means of three separate experiments ± SD. *$P < 0.05$, **$P < 0.01$, ***$P < 0.001$.

Supplementary Table S1: The sequences of primer used for ChIP-qRT-PCR

Primer name	Forward primer (5′-3′)	Reverse primer (5′-3′)
Primer 1	GACTGAATCTTGCTGAAGGGG	TTGACCAACCTGCCCATTAC
Primer 2	AAATCCACAGGTGCCAGAC	GAGGTCGCTTTATGCACTCTAT
Primer 3	GCACGTCGCTTCCTTTCTT	CACCATTGCGCTTCATCAC
Primer 4	CGAAGCCGTTGCCCTTT	GGAGCCAGGTTCTAGGG
Primer 5	CCTCGGATGGGAATTGGATAC	TGGACAGCACACAACCTATC

Supplementary Table S2: The sequences of primer used for qRT-PCR

Gene	Forward primer (5′-3′)	Reverse primer (5′-3′)
Hsa-miR-26b-5p	Order from GeneCopoeia:HmiRQP0357	Universal qPCR Primer (GeneCopoeia)
U6	Order from GeneCopoeia:HmiRQP0356	Universal qPCR Primer (GeneCopoeia)
CDH1	GAGTGCCAACTGGACCATTCAGTA	AGTCACCCACCTCTAAGGCCATC
VIM	TGACATTGAGATTGCCACCTACAG	TCAACCGTCTTAATCAGAAGTGTCC
TWIST1	GGCACCATCCTCACACCTCT	GCTGATTGGCACGACCTCT
SNAIL	GACCACTATGCCGCGCTCTT	TCGCTGTAGTTAGGCTTCCGATT
BMP4	TCCTCTTTAACCTCAGCAGCATC	GTCTCGTGTCCAGTAGTCGTGTG
SMAD1	ACAGTCTGTGAACCATGGATTTGA	TGAGGTGAACCCATTTGAGTAAGAA
GAPDH	GCACCGTCAAGGCTGAGAAC	TGGTGAAGACGCCAGTGGA

Supplementary Table S3: Antibodies used in this study

Antibody	Source	IHC Concentration Or WB	Product Number	Manufacture
E-cadherin	Mouse	1:50 (both)	Ab1416	Abcam
vimentin	Rabbit	1:200 (both)	ZA-0511	ZSCB-BIO
Smad1	Rabbit	1:2000 (both)	Ab63356	Abcam
Snail	Rabbit	1:200 (both)	Ab180714	Abcam
Slug	Rabbit	1:200 (only WB)	LS-B3449	LSBio
Bmp4	Rabbit	1:200 (only IHC)	Sc-9003	SANTA CRUZ
Bmp4	Rabbit	1:1000 (only WB)	Ab124715	Abcam
Twist1	Rabbit	1:50 (both)	Sc-15393	SANTA CRUZ
B-catenin	Rabbit	1:200 (both)	Ab32572	Abcam
Antibody	Source	Concentration(IF)	Product Number	Manufacture
E-cadherin	Mouse	1:50	Ab1416	Abcam
Vimentin	Rabbit	1:200	ZA-0511	ZSCB-BIO

Note: WB:Western blot.

Supplementary Table S4: Predicted targets of miR-26b-5p

Target	A	B	C	D	E	F	G	H	I
HGF	1	9	36	3	49	1	1	2	38.8
PRKCD	2	29	66	21	118	0	1	1	94.2
SMAD1	3	16	22	7	48	1	1	2	38
ULK2	4	8	3	12	27	0	0	0	21.6
FGD1	5	49	16	58	128	0	1	1	102.2
KCNJ2	6	48	26	20	100	0	1	1	79.8
ITGA5	7	57	30	22	116	2	1	3	92.2
PTEN	8	20	21	58	107	1	1	2	85.2
PLCB1	9	57	42	58	166	1	0	1	132.6
ATP1A2	10	31	95	43	179	0	2	2	142.8
VANGL2	11	21	19	58	109	1	1	2	86.8
CCDC6	12	19	14	58	103	1	1	2	82
UBE4B	13	12	32	33	90	0	0	0	72
MRAS	14	11	62	40	127	2	1	3	101
ACSL3	15	7	6	24	52	0	1	1	41.4
FRAT2	16	33	69	58	176	1	1	2	140.4
PRKCQ	17	35	71	58	181	0	0	0	144.8
RPS6KA6	18	1	38	17	74	1	1	2	58.8
SSX2IP	19	39	40	38	136	0	1	1	108.6
ULK1	20	4	13	6	43	0	0	0	34.4
SRGAP1	21	57	143	31	252	0	1	1	201.4
SSH2	22	57	39	34	152	1	1	2	121.2
NLK	23	57	24	2	106	1	1	2	84.4
DUSP5	24	57	143	29	253	2	2	4	201.6
CDK6	25	18	2	11	56	1	1	2	44.4
HUWE1	26	57	50	58	191	0	1	1	152.6
B3GNT5	27	2	43	58	130	0	0	0	104
ACVR1C	28	57	4	49	138	2	2	4	109.6
NTN4	29	57	143	45	274	2	1	3	218.6
ABL2	30	14	1	58	103	1	1	2	82
PTGS2	31	28	33	41	133	1	1	2	106
UBE2G1	32	26	18	8	84	0	1	1	67
BID	33	40	97	58	228	1	1	2	182
EIF2S1	34	30	9	58	131	0	1	1	104.6
INHBB	35	10	11	29	85	1	1	2	67.6
PAK2	36	57	12	54	159	2	1	3	126.6
MAN1A2	37	57	114	58	266	0	0	0	212.8
PIM1	38	50	65	28	181	0	1	1	144.6
HSPA8	39	43	93	58	233	0	1	1	186.2
PMAIP1	40	57	129	58	284	0	1	1	227
CACNB2	41	57	44	58	200	1	1	2	159.6

EPS15	42	54	28	58	182	2	1	3	145
RB1	43	41	88	58	230	0	1	1	183.8
RHOQ	44	3	15	58	120	0	0	0	96
DAPK1	45	57	84	23	209	0	1	1	167
BHLHE40	46	57	96	58	257	1	2	3	205
MAP3K2	47	57	31	10	145	1	1	2	115.6
LEF1	48	57	47	9	161	1	1	2	128.4
CREBBP	49	57	27	36	169	2	1	3	134.6
PSD3	50	57	5	58	170	0	1	1	135.8
YWHAE	51	57	112	32	252	1	1	2	201.2
CSNK1G1	52	57	8	58	175	2	0	2	139.6
COL1A2	53	47	117	37	254	1	1	2	202.8
MYH10	54	57	138	58	307	1	1	2	245.2
BCR	55	46	29	58	188	1	1	2	150
ACAP2	56	57	143	58	314	0	1	1	251
PI4K2B	57	5	133	58	253	1	1	2	202
GSK3B	58	25	10	5	98	1	1	2	78
B4GALT4	59	57	137	58	311	0	0	0	248.8
ERO1LB	60	57	35	4	156	0	0	0	124.8
CDC6	61	57	135	58	311	1	1	2	248.4
SHC4	62	57	89	58	266	1	1	2	212.4
CACNA1C	63	22	7	39	131	0	1	1	104.6
IPPK	64	57	25	58	204	0	0	0	163.2
PPP1R3D	65	57	139	58	319	0	1	1	255
PRKAG2	66	57	74	13	210	0	1	1	167.8
ADRBK2	67	57	59	58	241	1	1	2	192.4
UBE3A	68	57	82	58	265	0	1	1	211.8
PRKCB	69	57	143	58	327	1	1	2	261.2
PPP2R5A	70	57	110	58	295	0	0	0	236
SEMA6D	71	6	85	55	217	0	1	1	173.4
SAR1B	72	57	143	58	330	0	1	1	263.8
SMAD2	73	57	124	58	312	1	1	2	249.2
UBE2E2	74	57	111	25	267	0	1	1	213.4
STK4	75	57	54	58	244	1	1	2	194.8
RYR3	76	57	53	58	244	0	1	1	195
CCND2	77	57	46	56	236	2	1	3	188.2
PECAM1	78	57	45	58	238	1	1	2	190
EPHA7	79	57	81	52	269	1	1	2	214.8
GRIN2A	80	57	143	58	338	0	0	0	270.4
PIK3C2A	81	57	80	58	276	1	1	2	220.4
RAB31	82	57	140	58	337	0	1	1	269.4
UBE2D1	83	17	120	53	273	0	1	1	218.2
WNT5A	84	57	79	58	278	2	1	3	221.8
CDKN1C	85	57	68	58	268	0	1	1	214.2
ITGA4	86	57	101	58	302	1	1	2	241.2

UBE2K	87	32	121	58	298	0	1	1	238.2
MITF	88	57	143	58	346	0	1	1	276.6
DAB2	89	77	99	58	323	0	1	1	258.2
RAB11A	90	57	63	46	256	0	1	1	204.6
PRKAA2	91	57	104	58	310	0	1	1	247.8
RAP1A	92	45	130	35	302	0	1	1	241.4
COL11A1	93	57	98	58	306	1	1	2	244.4
ITPR1	94	57	102	58	311	0	0	0	248.8
SMAD4	95	57	86	48	286	1	1	2	228.4
ACVR2B	96	57	83	58	294	2	1	3	234.6
ARHGEF12	97	57	87	58	299	0	1	1	239
PPP3R1	98	15	103	51	267	1	1	2	213.2
ACSL4	99	57	56	58	270	0	1	1	215.8
SOX17	100	57	77	58	292	0	1	1	233.4
DNAJA2	101	57	64	26	248	0	1	1	198.2
COL5A1	102	57	75	14	248	1	1	2	198
PIK3R3	103	57	37	50	247	2	1	3	197
SH3KBP1	104	52	143	40	339	1	1	2	270.8
CACNB4	105	42	122	58	327	1	0	1	261.4
MAP3K7	106	57	143	58	364	2	1	3	290.6
ITGA6	107	57	72	19	255	1	1	2	203.6
MMP14	108	57	73	15	253	2	1	3	201.8
IGF1	109	57	94	58	318	1	1	2	254
WWP2	110	57	106	58	331	0	1	1	264.6
LTBP1	111	57	123	42	333	2	1	3	265.8
MAP3K1	112	57	131	58	358	0	1	1	286.2
DUSP4	113	57	126	58	354	2	2	4	282.4
E2F2	114	57	113	58	342	0	0	0	273.6
RPS6KA2	115	57	115	58	345	1	1	2	275.6
RORA	116	57	52	58	283	0	1	1	226.2

Note: A: miTG score, B: miRDB, C: Aggregate Pct, D: PicTar, E: A+B+C+D, F: EMT, G: KEGG signaling pathway, H: F+G, I: E*0.8+H*0.2.

MATERIALS AND METHODS

Chromatin immunoprecipitation (ChIP) assay

Detailed procedures for ChIP assay were previously described in [1]. Briefly, cells were cross-linked with 1% formaldehyde for 10 min, quenched with 0.125 M glycine, and lysed on ice for 10 min in cell swelling buffer containing 5 mM Pipes (pH 8.0), 85 mM KCl, 0.5% NP-40, 0.5 mM phenylmethylsulfonyl fluoride, and 100 ng/ml leupeptin and aprotinin. Nuclei were collected and resuspended in sonication buffer with 1% SDS, 10 mM EDTA, 50 mM Tris • HCl (pH 8.1), 0.5 mM phenylmethylsulfonyl fluoride, and 100 ng/ml leupeptin and aprotinin and incubated on ice for 10 min. Samples were sonicated to an average length of 0.5 kb and antibodies against Twist1 (Santa Cruz, USA), IgG (Santa Cruz, USA) were added to each aliquot of chromatin and incubated on a rotating platform overnight at 4°C. Antibody-protein-DNA complexes were isolated by immunoprecipitation with protein A agarose beads. Following extensive washing, bound DNA fragments were eluted and analyzed by subsequent qRT-PCR. Primer sequences are listed in the Table S1.

miRNA microarray analysis

miRNA microarray was performed by a service provider (LC Sciences). Total RNA (2 to 5 μg) from each sample was extracted using TRK1001 (LC Sciences, Cat. TRK-1001, Total RNA Purification Kit) according to the manufacturer's procedure. Total RNA was quantified by the NanoDrop ND-2000 (Thermo Scientific) and the RNA integrity was assessed using Agilent Bioanalyzer 2100 (Agilent Technologies). Labeling of total RNA was performed according to the protocol from LC Science for a dual-color fluorescence experiment with no modification. Hybridization was performed overnight on a μParaflo microfluidic chip using a micro-circulation pump (Atactic Technologies). On the microfluidic chip, each detection probe consisted of a chemically modified nucleotide coding segment complementary to target microRNA (from miRBase, http://www.mirbase.org/) or other RNA (control or customer defined sequences) and a spacer segment of polyethylene glycol to extend the coding segment away from the substrate. The detection probes were made by *in situ* synthesis using PGR (photogenerated reagent) chemistry. The hybridization melting temperatures were balanced by chemical modifications of the detection probes. Hybridization used 100 L 6xSSPE buffer (0.90 M NaCl, 60 mM Na2HPO4, 6 mM EDTA, pH 6.8) containing 25% formamide at 34°C. Fluorescence images were collected using a laser scanner (GenePix 4000B, Molecular Device) and digitized using Array-Pro image analysis software (Media Cybernetics). Data were analyzed by first subtracting the background, then the signals were normalized using a Lowess (locally weighted regression) filter. The raw microarray data set was filtered according to a standard procedure to exclude spots with minimum intensity. It was arbitrarily set to an intensity parameter of P100 for the miRNA microarray data. Spots with diameters less than 10 μm and flagged spots were also excluded from the analyses.

Description of in depth analysis

Multiple sample analysis included normalization, data adjustment, *t*-test/ANOVA analysis, and clustering [2]. A cyclic LOWESS (locally-weighted regression) method was used to carry out normalization [3]. Data adjustment included data filtering, $\log_2$ transformation, gene centering and normalization. Data filtering removed miRNAs with (normalized) intensity values below a threshold value of 32 across all samples. The $\log_2$ transformation converted intensity values into $\log_2$ scale. Gene centering and normalization transformed the $\log_2$ values using the mean and the standard deviation of individual genes across all samples using the following formula: value = [(value) – mean (gene)]/[standard deviation (gene)]. The *t*-test was performed between "control" and "test" sample groups with each group contains at least two samples [4]. *T*-values were calculated for each miRNA, and *p*-values were computed from the theoretical t-distribution. miRNAs with *p*-values below a critical *p*-value (typically 0.01) were selected for cluster analysis. The clustering was done using hierarchical method and was performed with average linkage and Euclidean distance metric [5]. All data processes except the clustering plot were carried out using in-house developed computer programs. The clustering plot was generated using TIGR MeV (Multiple Experimental Viewer) software from *The Institute for Genomic Research*.

REFERENCES

1. Liu Z, Garrard WT. Long-range interactions between three transcriptional enhancers, active Vkappa gene promoters, and a 3´ boundary sequence spanning 46 kilobases. Mol Cell Biol. 2005; 25:3220–3231.
2. Li Y, Wang F, Xu J, Ye F, Shen Y, Zhou J, Lu W, Wan X, Ma D, Xie X. Progressive miRNA expression profiles in cervical carcinogenesis and identification of HPV-related target genes for miR-29. J Pathol. 2011; 224:484–495.
3. Bolstad BM, RAI M, Astrand TP, Speed. A comparison of normalization methods for high density oligonucleotide array data based on variance and bias. BIOINFORMATICS. 2003; 19:185–193.
4. Pan W. A comparative review of statistical methods for discovering differentially expressed genes in replicated microarray experiments. Bioinformatics. 2002; 18:546–554.
5. Eisen MB, Spellman PT, Brown PO, Botstein D. Cluster analysis and display of genome-wide expression patterns. Proc Natl Acad Sci U S A. 1998; 95:14863–14868.
